# Basal procalcitonin, C-reactive protein, interleukin-6, and presepsin for prediction of mortality in critically ill septic patients: a systematic review and meta-analysis

**DOI:** 10.1186/s41512-023-00152-2

**Published:** 2023-08-03

**Authors:** Daniel Molano-Franco, Ingrid Arevalo-Rodriguez, Alfonso Muriel, Laura del Campo-Albendea, Silvia Fernández-García, Ana Alvarez-Méndez, Daniel Simancas-Racines, Andres Viteri, Guillermo Sanchez, Borja Fernandez-Felix, Jesus Lopez-Alcalde, Ivan Solà, Dimelza Osorio, Khalid Saeed Khan, Xavier Nuvials, Ricard Ferrer, Javier Zamora, the SEPSIS-BIOMARKERS Collaborators, Alvaro Estupiñan, Luis Franco, Jorge Cardenas, Ivan Robayo, Mario Villabon, Mario Gomez, Elena Stalling, Noelia Alvarez

**Affiliations:** 1 Hospital San José, Fundación Universitaria de Ciencias de la Salud (FUCS), CIMCA Research Group, Bogotá, Colombia; 2 Clinical Biostatistics Unit, Hospital Universitario Ramón y Cajal, IRYCIS, CIBER of Epidemiology and Public Health (CIBERESP), Madrid, Spain; 3 Nursing and Physiotherapy Department, University of Alcala, Madrid, Spain; 4 WHO Collaborating Centre for Global Women’s Health, Institute of Metabolism and Systems Research, University of Birmingham, Birmingham, UK; 5 Nursing Department, Faculty of Nursing, Physiotherapy and Podiatry, Complutense University of Madrid, Madrid, Spain; 6 Centro de investigación en Salud Pública y Epidemiología Clínica (CISPEC) Facultad de Ciencias de la Salud “Eugenio Espejo”, Universidad UTE, Quito, Ecuador; 7 Hospital Universitario Mayor-Méderi; Universidad del Rosario, Bogota, Colombia; 8 Universidad Francisco de Vitoria, Pozuelo de Alarcon, Spain; 9 Institute for Complementary and Integrative Medicine, University Hospital Zurich and University of Zurich, Zurich, Switzerland; 10 Iberoamerican Cochrane Centre, IIB SANT PAU, CIBER of Epidemiology and Public Health (CIBERESP), Barcelona, Spain; 11 Health Services Research Group, Vall d’Hebron Institut de Recerca (VHIR), Vall d’Hebron Hospital Universitari, Vall d’Hebron Barcelona Hospital Campus, CIBER of Epidemiology and Public Health (CIBERESP), Barcelona, Spain; 12 Department of Preventive Medicine and Public Health, Faculty of Medicine, University of Granada, CIBER of Epidemiology and Public Health (CIBERESP), Granada, Spain; 13 Critical Care Department, Vall d’Hebron University Hospital, Shock Organ Dysfunction and Resuscitation Research Group (SODIR), Vall d’Hebron Research Institute (VHIR), Barcelona, Spain; 14 Institute of Metabolism and Systems Research, University of Birmingham, Birmingham, UK

**Keywords:** Sepsis, Mortality, Biomarkers, Prognostic factors, Systematic review

## Abstract

**Background:**

Numerous biomarkers have been proposed for diagnosis, therapeutic, and prognosis in sepsis. Previous evaluations of the value of biomarkers for predicting mortality due to this life-threatening condition fail to address the complexity of this condition and the risk of bias associated with prognostic studies. We evaluate the predictive performance of four of these biomarkers in the prognosis of mortality through a methodologically sound evaluation.

**Methods:**

We conducted a systematic review a systematic review and meta-analysis to determine, in critically ill adults with sepsis, whether procalcitonin (PCT), C-reactive protein (CRP), interleukin-6 (IL-6), and presepsin (sCD14) are independent prognostic factors for mortality. We searched MEDLINE, EMBASE, and the Cochrane Central Register of Controlled Trials up to March 2023. Only Phase-2 confirmatory prognostic factor studies among critically ill septic adults were included. Random effects meta-analyses pooled the prognostic association estimates.

**Results:**

We included 60 studies (15,681 patients) with 99 biomarker assessments. Quality of the statistical analysis and reporting domains using the QUIPS tool showed high risk of bias in > 60% assessments. The biomarker measurement as a continuous variable in models adjusted by key covariates (age and severity score) for predicting mortality at 28–30 days showed a null or near to null association for basal PCT (pooled OR = 0.99, 95% CI = 0.99–1.003), CRP (OR = 1.01, 95% CI = 0.87 to 1.17), and IL-6 (OR = 1.02, 95% CI = 1.01–1.03) and sCD14 (pooled HR = 1.003, 95% CI = 1.000 to 1.006). Additional meta-analyses accounting for other prognostic covariates had similarly null findings.

**Conclusion:**

Baseline, isolated measurement of PCT, CRP, IL-6, and sCD14 has not been shown to help predict mortality in critically ill patients with sepsis. The role of these biomarkers should be evaluated in new studies where the patient selection would be standardized and the measurement of biomarker results.

**Trial registration:**

PROSPERO (CRD42019128790).

**Supplementary Information:**

The online version contains supplementary material available at 10.1186/s41512-023-00152-2.

## Introduction

Sepsis, a life-threatening organ dysfunction resulting from a dysregulated host response to an infection [1], occurs in one per 1000 people worldwide and accounts for 10% of intensive care unit (ICU) admissions [2]. Its complications include acute renal failure, polyneuropathy, cardiomyopathy, and multiple organ dysfunction [3, 4]. Mortality in septic shock can be > 40% [1]. About 50–70% of sepsis survivors suffer persistent physical, psychological, mental, and social issues [5, 6]. Management of sepsis is challenging, and it may include early administration of antibiotics, restoring tissue perfusion by resuscitation with crystalloids and vasopressors, and controlling the infection source [3, 7]. Care needs to be individualised, with stratification of patient’s mortality risk [8, 9].

Biomarkers have been proposed as the key to tailoring therapies for specific patients and monitoring their effects [10]. Currently, more than 150 potential sepsis biomarkers have been described [11]; however, many of them correspond to the measurement of substances derived from the complex process that sepsis represents, in its microorganism-host interaction, without its clinical utility being determined to date. Four of these biomarkers: procalcitonin (PCT), C-reactive protein (CRP), interleukin (IL)-6, and presepsin (sCD14) are commonly assessed biomarkers, and they have been evaluated for diagnosis and prognosis purposes [8, 12–15]. With respect to sepsis prognosis, single biomarkers or their various combinations may be useful in estimating the risk of death, re-hospitalisation, or long-term complications as they can help detect endothelial damage, intestinal permeability and organ failure early [15, 16]. Biomarker results may be added to clinical scores to improve prediction. The timing of measurement and the kinetics of their clearance are key points in the prognostic investigation [17]. However, conclusions about their value are not firm. Although the Surviving Sepsis Campaign guidelines for the management of sepsis consider biomarkers as an add-on of clinical evaluation, in the Sepsis-3 definition consensus their role remains still undefined [1].

Beyond the well-established role of some biomarkers for the diagnosis and management of septic patients, a formal assessment of the prognostic role of biomarkers in the prediction of critical sepsis outcomes using a methodologically sound critical appraisal is lacking [18]. The objective of this systematic review was to determine whether PCT, CRP, IL-6, and sCD14 are independent prognostic factors for predicting mortality in critically ill adults with sepsis.

## Materials and methods

### Search strategy and selection criteria

We conducted this systematic review and meta-analysis following recommended methods [18, 19]. The review was prospectively registered (PROSPERO, CRD42019128790) and reported following the PRISMA (Preferred Reporting Items for Systematic reviews and Meta-Analysis) statement [20]. We searched MEDLINE-Ovid, EMBASE-Ovid, and the Cochrane Central Register of Controlled Trials (CENTRAL) using a sensitive search strategy without language restrictions, from inception date to March 24 2023 (Additional file S1). We also hand-searched the reference lists of included studies.

Pairs of reviewers independently screened titles, abstracts, and then full-text articles. Disagreements were resolved by discussion with a third reviewer. We selected for this review phase 2 confirmatory prognostic factor studies [21], which measure a factor’s independent prognostic effect while controlling for known covariates. Studies considered phase 1 exploratory studies (i.e., those identifying potential associations or differences without an adequately adjusted analysis), as well as case series, diagnostic accuracy studies, and other studies not focussed on prognosis were excluded.

Studies were eligible for inclusion if they (a) included at least ten patients and at least 5 events; (b) included adults (males and females aged ≥ 18 years) reported as being critically ill and with a confirmed diagnosis of sepsis following valid clinical criteria; (c) measured at least one of the four biomarkers under assessment within the first 24 h of sepsis diagnosis or admission to the intensive care unit, emergency department or hospital ward; (d) reported any measure of patient’s survival (such as mortality or survival), ideally at 28–30 days following hospitalisation. If a study reported similar patients from the same institution in more than one publication, we included the paper with the largest sample in our analysis to avoid spurious precision due to duplication.

### Data analysis

Pairs of reviewers independently extracted the following data from each eligible study, using a standardised form: study general characteristics, including number and name of centres involved, study funding and declaration of conflict of interests; population characteristics, including criteria for sepsis diagnosis, age and sex, severity scores (e.g., SOFA, APACHE, SAPS) and hospital setting (e.g., ICU, emergency room); biomarkers characteristics, including the timing and unit of measurement; and mortality reports, including the number of survivors/deaths and the corresponding proportions. Study authors were contacted for clarifications when necessary.

The primary outcome of this systematic review was mortality. This outcome included all-cause mortality, sepsis-related mortality, ICU or hospital mortality, ideally measured at 28–30 days, as defined by the study authors. For statistical analysis, we classified all measurements performed at 28–30 days as “mortality at 28–30 days” (i.e., hospital mortality at 28–30 days, ICU mortality at 28–30 days, survival at 28–30 days and sepsis-related mortality at 28–30 days); other reports without a clear statement about the follow-up duration (i.e., hospital mortality, ICU mortality, survival/non-survival) were classified as “mortality-no details”.

We assessed the methodological quality of the included studies using the Quality In Prognosis Studies (QUIPS) tool, which contains six domains [22] that have been tailored for this review (see Additional file S2). Clinical experts participating in these reviews selected a list of prognostic covariates to be considered in the QUIPS assessment. These covariates were age, a severity score (e.g., SOFA, SAPS, or APACHE), use of vasopressors, key comorbidities (i.e., immunosuppression, pulmonary disease, cancer, alcohol dependence, lymphocytopenia), inappropriate empirical antibiotic regimen, late antibiotic coverage and control of septic focus. Age and severity score were regarded as the key covariates that predictive models should include for a minimal adequacy of the adjustment (QUIPS Domain 5). Pairs of reviewers independently assessed the risk of bias. Clarifications were requested from the study authors when necessary. We made a consensus judgement for each of the 6-QUIPS domains, using one of the three risk of bias categories (i.e., low, moderate or high) suggested by the tool guidelines [18].

We extracted information regarding the predictive value of each biomarker, including the baseline measurement of survival and no-survival groups (e.g., medians and interquartile ranges, IQRs), the corresponding effect measure derived from a multivariable model (e.g., adjusted odds ratio, OR, with 95% confidence interval, CI). If the authors had categorized the underlying continuous variables of the biomarkers, we extracted the proposed positivity threshold (i.e., the biomarker cut-off proposed by the authors to predict mortality).

Due to the biomarkers being measured in different units across studies (e.g., mg/L, g/mL, µg/L), we transformed the numerical information to the most frequent unit reported for the biomarker, as follow: PCT: ng/ml; CRP: mg/L; IL-6: pg/mL; and sCD14: pg/mL. Studies in which the biomarker was transformed into a logarithmic scale were analysed separately because a transformation to a common unit was not possible. We cross-checked the accuracy of the numerical findings using all the additional numerical information provided in the reports, such as the p-values, the CIs and Wald’s test results, and clarifications were requested from the study authors when necessary. In cases where the study authors did not evaluate the biomarker in their final model but provided information from univariable analysis (e.g., an initial univariable logistic model), we extracted this information as a proxy for the performance of the biomarker in a multivariable analysis as a last resource. In cases where only narrative information about the biomarker was available, we extracted the reported “statistically significance” of the association (commonly based on p-values) and added it narratively to our synthesis. We prioritised as main analysis the association of the biomarker measured as a continuous variable with “mortality at 28–30 days” in models adjusted for the covariates age and severity score based on the consensus of our expert panel.

We aggregated data within clinical and statistical relevant groups by each biomarker using a random effects restricted maximum likelihood (REML) meta-analysis model, providing pooled estimates (hazard ratio or OR) according to the Sidik-Jonkman method, along with the corresponding Hartung-Knapp 95% CI and between-study variance estimates. To account for the heterogeneity in the pooled effects, we calculated also the 95% prediction interval. All statistical analyses were performed using Stata 17.

## Results

**Fig. 1:**
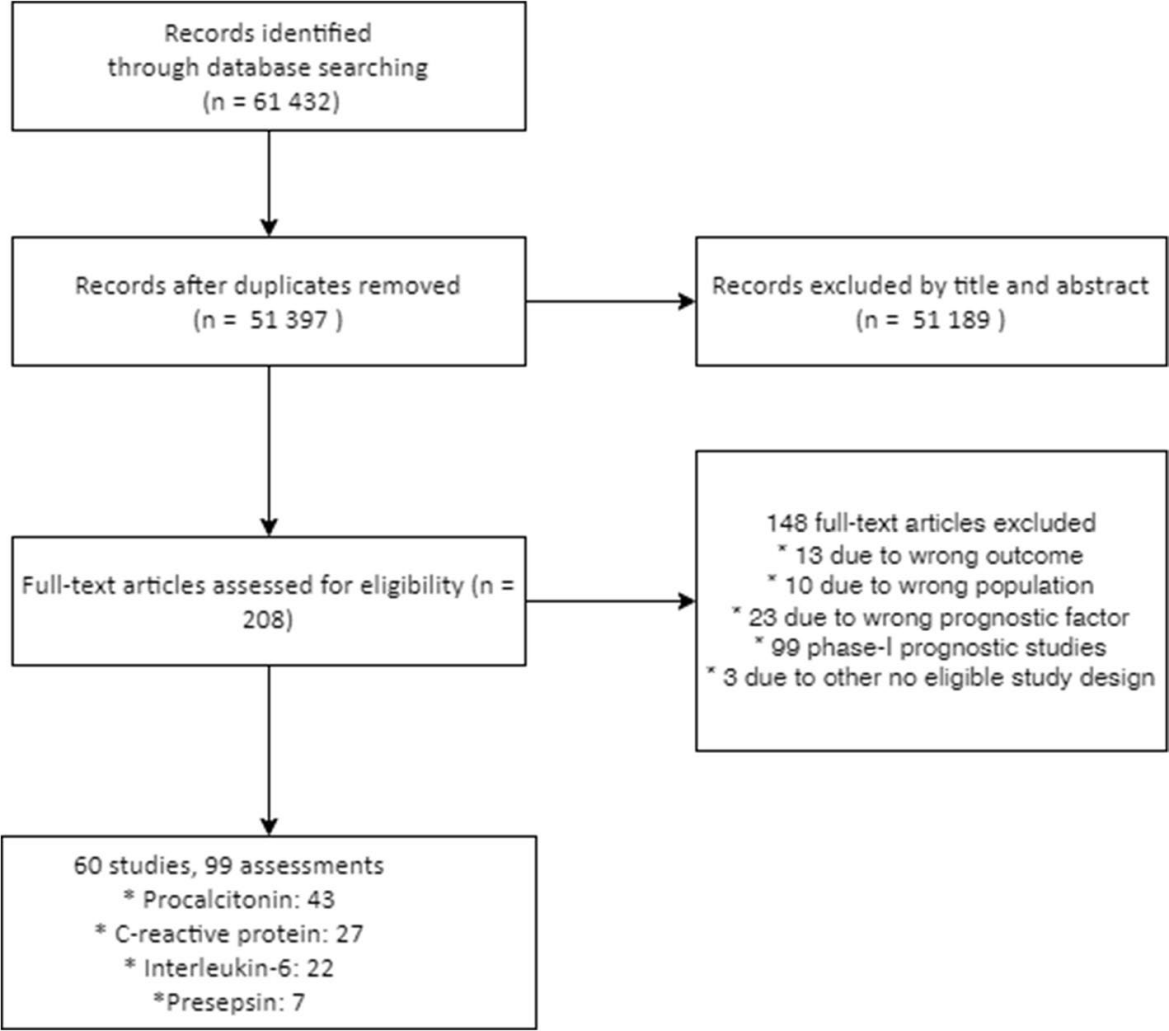
Flow diagram of studies selection in the systematic review of prognostic value of biomarkers among critically ill adults with sepsis

After removing duplicates, we identified 61 432 records in electronic database searches (Fig. 1). After excluding 51,189 records at the title and abstract level, 208 full texts were screened. Forty-nine publications were excluded due to ineligible participant sample (undefined mix of septic and no-septic patients), prognostic factor, outcome, or study design (Additional file S3). Another 99 studies were excluded as they were exploratory studies, i.e., those reporting differences between survivors and non-survivors. Finally, 60 studies that met our inclusion criteria and provided 99 biomarker assessments (Fig. 1) evaluating the prognostic role of PCT (43 assessments), CRP (27 assessments), IL-6 (22 assessments), and sCD14 (7 assessments).

Sixty studies including 15,681 critically ill septic patients evaluated the role of at least one of our selected biomarkers for mortality prediction [23–62]. We contacted 11 study teams for additional information, and we received replies from three [27, 33, 56]. Fifty-five (91.7%) studies were published in the last 10 years (2012–2023), and 30 (50.0%) were published in the previous 3 years. Studies were primarily performed in China (28 studies), South Korea (6) and Spain (4). Most of the studies involved patients from a single centre (49 studies, 81.7%), intensive care unit (ICU) being the most frequent hospital setting (41 studies, 68.3%). Only one study comprised information from two study centres (two ICUs from Spain and France). Fifty-three studies (88.3%) were cohorts, three analysing data from a randomised controlled trial. One study team reported information about a similar set of patients from the same institution in two different publications [32, 63]; we included the data form the report with the largest sample size (187 patients versus 160). Study funding was mainly public (22 studies, 36.7%), with 20 studies not reporting this information. Authors of 44 studies (73.3%) declared no conflict of interest (Additional file S4).

Regarding the population under analysis, the sample size ranged from 29 [25] to 1984 [64] septic patients, with a median of 145 (25th–75th percentiles 89–267). The percentage of women ranged from 23.7% to 67%. Studies that reported mean age for septic patients found the range from 47.5 to 84.3 years (39). Diagnosis of sepsis was primarily performed using the 2015 SCCM/ESICM Sepsis-3 (1) (30 studies, 50.0%) and the 2001 SCCM/ESICM/ACCP/ATS/SIS [7] (16 studies, 26.7%). In some cases, authors used a specific definition of a septic condition (i.e., community-acquired infection) or local guidelines about sepsis. Sepsis origin was mixed in 71.7% of populations (i.e., combining respiratory, urogenital, intra-abdominal, blood and skin-soft tissues, among others). One study excluded patients under mechanical ventilation, [55] and two excluded patients using vasopressors [32, 55]. Only three studies reported the use of empirical antibiotics, being in both cases used in more than 90% of patients [48, 54, 65].

Regarding the assessment of biomarkers, 41/60 studies (68.3%) evaluated a single biomarker in their analysis. PCT was the most frequently assessed biomarker (40 studies and 43 assessments). Mortality rate across the studies ranged from 7 to 65%. Eighty-two assessments addressed the relationship between one biomarker and the mortality at 28–30 days (87.2%), while the remaining assessments focused on mortality without further details, including hospital and ICU mortality (Table 1).

**Table 1:** Descriptive summary of studies included in the systematic review of prognostic value of biomarkers among critically ill adults with sepsis

**Study characteristics**	
Year of publication; *n* (%)	
≤2015	16 (27)
2016–2022	44 (73)
Number of centres involved; *n* (%)	
Single centre	49 (81.7)
Two centres	3 (5.0)
4 or more centres	8 (13.3)
Participants’ recruitment in the study; *n* (%)	
Prospective	44 (73.3)
Retrospective	16 (26.7)
Study design;* n* (%)	
Cohort	54 (90.0)
Case-control	6 (10.0)
Study funding (not reported = 20 studies); *n* (%)	
Public	22 (36.7)
Private/industry	3 (5.0)
Mixed	4 (6.7)
No funding	11 (18.3)
Conflict of interest. (not reported = 13 studies); *n* (%)	
No conflicts	44 (73.3)
Conflicts reported	3 (5.0)
**Population characteristics**	
Sample size; median (25th 75th percentiles)	145; 5 (89 to 267)
Age (years) (Not reported = 1 study); *n* (%)	
Means (min.—max.)	40.5 to 84.3
Medians (min.—max.)	32 to 79
Sex-female (min.—max)	23.7% to 67%
Diagnosis of sepsis. (not reported = 3 studies); *n* (%)	
ACCP/SCCM 1991	3 (5.0)
2001 SCCM/ESICM/ACCP/ATS/SIS	16 (26.7)
2015 SCCM/ESICM Sepsis-3	30 (50.0)
Other criteria	6 (10.0)
Septic shock only; n (%)	17 (28.3)
Sepsis origin (Not reported = 13 studies); *n* (%)	
Mixed	43 (71.7)
Respiratory only	3 (5.0)
Bacteraemia only	1 (1.7)
APACHE scores (not reported = 25 studies)	
Means (min.—max.)	3.6 to 32.3
Medians (min.—max.)	15 to 33
SOFA scores (not reported = 18 studies)	
Means (min.—max.)	2.3 to 29.7
Medians (Min. — Max.)	4 to 11
SAPS-II scores (not reported = 53 studies)	
Means (min.—max.)	22.1 to 83.6
Medians (min.—max.)	41 to 63
Report of comorbidities; *n* (%)	30 (50.0)
Hospital setting; *n* (%)	
Emergency room	16 (26.7)
Intensive care unit	41 (68.3)
Mixed	3 (5.0)
Mechanical ventilation (not reported = 46 studies), (min.—max.)	19.9% to 98.3%
Use of vasopressors (not reported = 51 studies), (min.—max.)	4.5% to 91.1%
Patients on dialysis (not reported = 56 studies), (min.—max.)	22% to 68%
Duration of ICU stay/days (not reported = 29 studies)	
Medians (min.—max.)	4 to 15.5
**Biomarkers characteristics**	
Number of biomarkers by study (*n* = 60 studies); *n* (%)	
One biomarker assessment	32 (53.3)
Two biomarkers assessments	23 (38.3)
3 or 4 biomarkers assessments	5 (3.4)
Procalcitonin (40 studies, 43 assessments); *n* (%)	
Timing of measurement	
At admission	15 (37.5)
Within 24 h of admission	15 (37.5)
At the time of diagnosis	8 (20.0)
Not reported	2 (5.0)
Unit of measurement	
ng/mL	28 (70.0)
µg/L	4 (10.0)
pg/mL	1 (2.5)
g/mL	1 (2.5)
ng/dL	1 (2.5)
mg/L	1 (2.5)
g/L	1 (2.5)
log─ng/mL	2 (5.0)
log─µg/mL	1 (2.5)
C-reactive protein (25 studies, 27 assessments); *n* (%)	
Timing of measurement	
At admission	10 (40.0)
Within 24 h of admission	10 (40.0)
At the time of diagnosis	4 (16.0)
Not reported	1 (4.0)
Unit of measurement	
mg/L	13 (52.0)
mg/dL	8 (32.0)
pg/mL	1 (4.0)
log─mg/L	2 (8.0)
log─mg/dL	1 (4.0)
Interleukin-6 (22 studies, 22 assessments); *n* (%)	
Timing of measurement	
At admission	6 (27.3)
Within 24 h of admission	11 (50.0)
At the time of diagnosis	3 (13.6)
Not reported	2 (9.1)
Unit of measurement	
pg/mL	16 (72.7)
ng/mL	1 (4.5)
ng/L	1 (4.5)
log─pg/mL	4 (18.3)
Presepsina (6 studies, 6 assessments); *n* (%)	
Timing of measurement	
At admission	2 (33.3)
Within 24 h of admission	3 (50.0)
Not reported	1 (16.7)
Unit of measurement	
pg/mL	4 (66.6)
µg/mL	1 (16.7)
log─pg/mL	1 (16.7)
**Outcome characteristics**^**a**^	
Min–max mortality (%)	7.04 to 65.3
No reported/not estimable	Five studies
Mortality at 28–30 days (51 assessments); *n* (%)^a^	
Mortality during the first 28–30 days, no details provided	40 (78.4)
Hospital mortality during the first 28–30 days	4 (7.8)
Sepsis-related mortality measured at 28–30 days	4 (7.8)
ICU mortality during the first 28–30 days	3 (6.0)
Mortality (general, no details) (10 assessments); *n* (%)^a^	
Hospital mortality (no-survival), no details provided	6 (50.0)
ICU mortality (no-survival), no details provided	5 (50.0)

^a^Percentages could be greater than 100% as the same assessment could have use more than one outcome

Across all biomarkers, the QUIPS domain most affected by risk of bias was the statistical analysis and reporting domain (Domain 6), with > 60% of assessments at high risk of bias, mostly due to inadequate reporting of the final model (i.e., report of statistically significant values only), optimal cut-off used to estimate the final effect measure and impossibility to reproduce the confidence intervals using the standard errors and coefficients provided in the report. The study participation domain (Domain 1) was judged as low risk of bias for three of the four biomarkers, with more than 50% of assessments considered with an adequate participation of the eligible participants, a clear reporting of inclusion/exclusion criteria, and in some cases, a provision of a flow diagram. Study confounding domain (Domain 5) was commonly affected because important key prognostic covariates were not accounted in the analysis. Due to the poor reporting of confounders in the included studies, we considered only age and severity score (i.e., SOFA, SAPS or APACHE) as the minimal set of prognostic factors for the multivariable analysis. We found 31 assessments with these prognostic factors being considered in the final statistical models and so, they were considered at low risk of bias (31%). The remaining domains were considered at moderate risk of bias, mostly due to unclear reporting of missing participants (i.e., losses at follow-up), missing information (i.e., missing biomarker information), and unclear methods of measurement (i.e., mortality measurement). Only two study providing information about PCT, IL-6 and CRP was considered at low risk of bias in all QUIPS-domains (Additional file S5) [62, 66].

We found a heterogeneity in the analysis and reporting of the association between biomarkers and mortality from the included studies. Baseline biomarker levels were analysed as a continuous variable in 64 assessments (65%), 13 of them being analysed after a logarithmic transformation. In 23 assessments, authors omitted the report of the effect measure, mostly due the analyses were not significant either in the univariable or the multivariable analysis. In the remaining assessments, the study authors chose a threshold, mostly derived as the optimal cut-off, and analysed the biomarker as a categorical variable. We report here the findings of biomarkers as a continuous variable in prognostic models accounting for age and severity score as confounding factors in relation to mortality at 28–30 days. Detailed information regarding the remaining assessments from included studies (e.g., categorical, log- transformed and narrative reports), as well as the information for other mortality outcomes (i.e., mortality-general, no details), is shown in the Additional files S5–S8 and Additional figs. S1–S5.

**Fig. 2:**
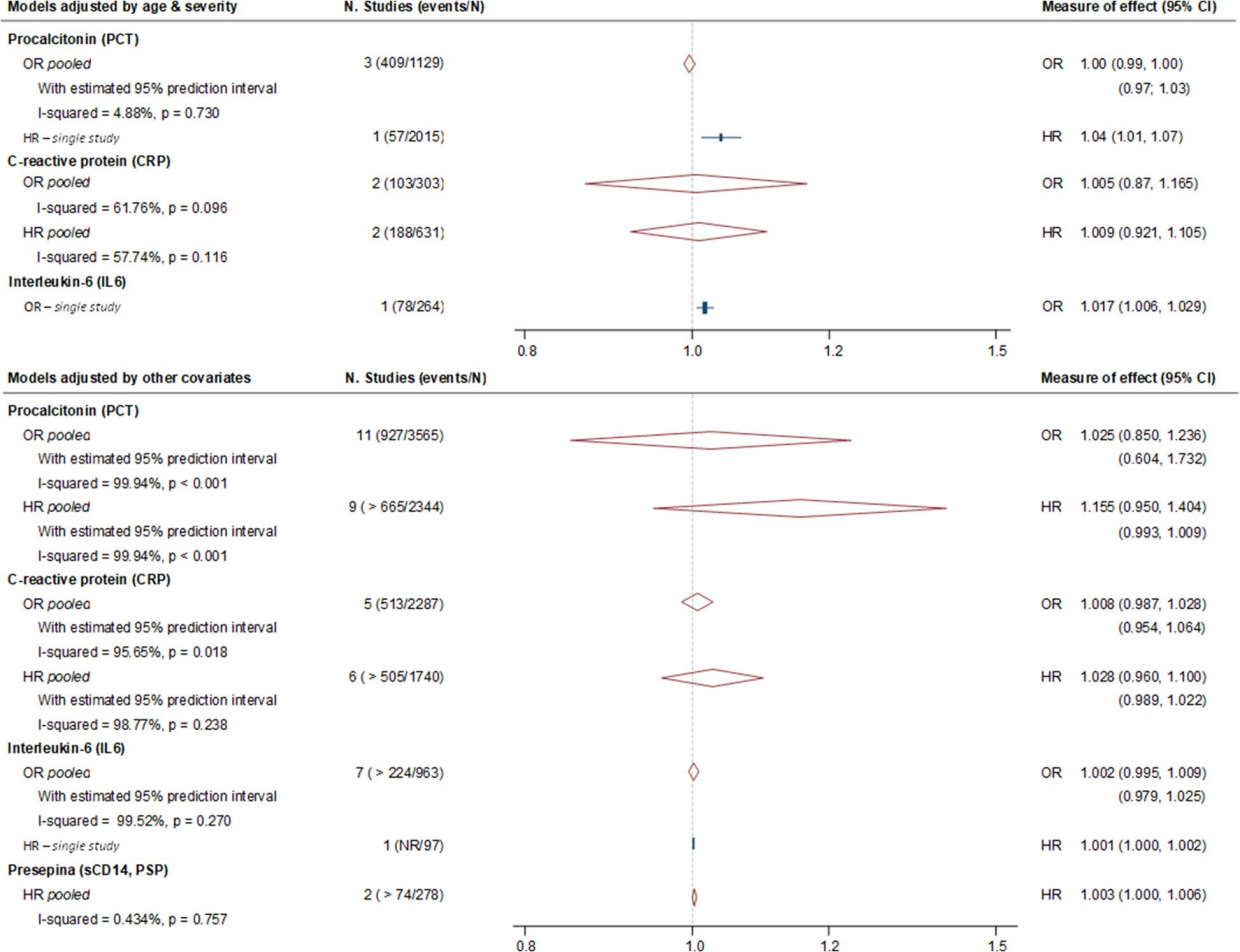
Relation of baseline biomarkers measures with mortality at 28–30 days: selected biomarker performance assessments

Regarding basal PCT (ng/ml), forty out of 94 assessments focused on baseline PCT levels and its prognostic value for mortality, mortality at 28–30 days being the outcome most reported (35/43, 81.4%). Analysis focus on studies accounting by age and a severity score as a confounding factors, showed a null association between basal PCT and mortality at 28–30 days (pooled OR = 0.99, 95% CI = 0.99 to 1.00, 95% prediction interval = 0.97 to 1.03; *I*^2^ = 4.9%; 3 studies, 1129 patients) (Fig. 2, Additional Table S6, Additional Fig. S1). Only one study accounted by age and a severity in a multivariable cox regression model, and it showed a significant association (HR = 1.04, 95% CI = 1.01 to 1.07, 1 study, 2015). Additional pooled analysis without this minimal adjustment showed similar findings, either using OR (pooled OR = 1.02, 95% CI = 0.85 to 1.24, 95% prediction interval = 0.60 to 1.73; *I*^2^ = 99.9%; 11 studies, 3565 patients) or HR measures of effect (pooled HR = 1.16, 95% CI = 0.95 to 1.40, 95% prediction interval = 0.99 to 1.01; *I*^2^ = 99.9%; 9 studies, > 2344 patients) (Fig. 2, Additional Table S6, Additional Fig. S1). Most of the remaining assessments derived from categorical, log-transformed variables and narrative reports showed no role for PCT in the prognosis of mortality (Additional file S6).

Regarding Basal CRP (mg/L), twenty-five out of 94 assessments focused on baseline CRP levels and its association with mortality, mortality at 28–30 days being the most frequent outcome (21/25, 84.0%). Analysis focus on studies accounting for age and a severity score as a confounding factors, showed a null association between basal CRP and mortality at 28–30 days in multivariable logistic and Cox regression models (pooled OR = 1.01, 95% CI = 0.87 to 1.17, *I*^2^ = 61.8%, 2 studies, 303 patients; pooled HR = 1.01, 95% CI = 0.92 to 1.11, *I*^2^ = 57.7%, 2 studies, 631 patients) (Fig. 2, Additional Fig. S2, Additional Table S7). Pooled analysis without this minimal adjustment showed similar findings, either using OR (pooled OR = 1.01, 95% CI = 0.99 to 1.03, 95% prediction interval = 0.95 to 1.06, *I*^2^ = 95.7%; 5 studies, 2287 patients) or HR measures of effect (pooled HR = 1.03, 95% CI = 0.96 to1.10, 95% prediction interval = 0.99 to1.02; *I*^2^ = 98.8%; 6 studies, > 1741 patients) (Fig. 2, Additional Fig. S2). Most of the remaining assessments derived from categorical, log-transformed variables and narrative reports showed no role for CRP in the prognosis of mortality (Additional Table S7).

Regarding basal IL-6 (pg/ml), 22 out of 94 assessments were focused on baseline IL-6 levels and its association with mortality, mortality at 28–30 days being the most frequent outcome (20/22, 90.9%). Only one study accounted by age and a severity score as a confounding factors, and it showed a near to null association between basal IL-6 and mortality at 28–30 days (OR = 1.02, 95% CI = 1.01 to1.03) (Fig. 2, Additional Fig. S3, Additional Table S8). Pooled analysis without this minimal adjustment showed similar findings (pooled OR = 1.00, 95% CI = 0.99 to 1.01, 95% prediction interval = 0.98 to 1.02; I-square = 99.5%; 7 studies, > 963 patients; HR = 1.00, 95% CI = 1.00 to 1.00, 1 study, 97 patients) (Fig. 2, Additional Fig. S3). Most of the remaining assessments derived from categorical, log-transformed variables and narrative reports showed no role for IL-6 in the prognosis of mortality (Additional Table S8).

Regarding basal sCD14 (pg/ml), only six assessment focused on baseline sCD14 levels and prediction of mortality (Additional Table S9). Only two assessments accounting by key prognostic factors and analysing the biomarker as a continuous variable without minimal adjustment. This analysis shows a significant relationship between sCD14 levels, although the effect size is small (pooled HR = 1.00, 95% CI = 1.00 to 1.01; *I*^2^ = 0.4%; 2 studies, > 278 patients. Information from categorical and log-transformed analysis is contradictory for the association between sCD14 and mortality, including 28–30 days (Additional Table S9).

A sensitivity analysis combining information of mortality categories only affect selected pooled estimations of PCT and CRP levels. However, the measures of effect estimated combining these assessments, as well as the corresponding 95% confidence intervals, did not show an increased risk of mortality with any of these biomarkers, similar to the findings using separate mortality categories.

## Discussion

This systematic review, comprising of studies mostly published in the last decade (2011–2023), found a lack of association between the baseline values of PCT, CRP, IL-6, and sCD14 and the risk of mortality, especially at 28–30 days, in critically ill septic patients diagnosed using current criteria. This evidence covered information from several countries and teams worldwide and recruited a large number of septic patients. Quality of the evidence was affected by several sources of bias; the QUIPS domain most affected being the statistical analysis and reporting (Domain 6) across all biomarkers. This deficiency limited the strength of the inferences drawn.

In general, the number of assessments showing no association between the baseline biomarker values and mortality was greater than those showing a positive association. When we focussed on assessments of the biomarker as a continuous variable and adjusted by key covariates for the optimal prognostic evaluation, we found a null association of basal PCT and CRP with mortality at 28–30 days (For PCT = pooled OR = 0.99, 95% CI = 0.99–1.003, 95% prediction interval (PI) = 0.97–1.03; *I*^2^ = 4.9%; 3 studies, 1129 patients. For CRP = pooled OR = 1.01, 95% CI = 0.87 to 1.17, *I*^2^ = 61.8%, 2 studies, 303 patients). For IL-6, we only found individual assessment accounting for these key covariates and showed also null or close to null association for mortality 28–30 days prediction (For IL-6: OR = 1.02, 95% CI = 1.01–1.03). However, additional pooled analysis accounting for other prognostic covariates are in agreement with these findings (see Additional Tables S6–S9). Regarding SCD14 assessment, we only found information derived from six assessments with contradictory findings. These assessments are mostly from log-transformed data, which prevented their aggregation with other untransformed estimates to avoid compromising the clinical interpretability of the pooled estimates [67].

Our study has several strengths. Firstly, our systematic review involved a comprehensive search of the literature, with more than 60,000 hits identified from electronic searches. We identified more evidence regarding the prediction of mortality at 28–30 days, which is a clinically important outcome for managing patients adequately according to their levels of basal risk. We made extra efforts to confirm the accuracy of the numerical data and standardised the units of measurement to able a pooled data analysis. Although we made efforts to unify the information to synthesize the data, the variability of reporting was the main constraint. In addition, our review follows the current recommended methodology to conduct systematic reviews to evaluate prognostic factors, selecting information from phase 2 confirmatory prognostic studies using multivariable models and adjustment for confounding [18, 68]. Other non-systematic reviews regarding the prognosis of mortality in sepsis have focused on exploratory data with no control for confounding (i.e., the merely compute differences in means between survivors and non-survivors) and claim these biomarkers’ role in the clinical setting without a discussion about the limitations [15, 16].

Limitations of our findings include the risk of bias affecting the included studies, primarily due to the lack of report of critical statistical and clinical information (e.g., the full results of the multivariable model). Although we asked for clarifications from the study authors, this information was dismissed without proper registration in some cases. We also noticed that control of confounding was not optimal in several analyses, and models failed to incorporate the key set of prognostic confounders, such as age and a severity score. The sample size of the studies was also a concern, as in some studies it led to unreliable estimates from the multivariable models. Future studies, to be considered confirmatory (i.e., phase 2) should include a minimal set of covariates while assessing the prognostic value of factors in the sepsis scenario. Regrettably, the limited availability of valid information for analysis hindered our ability to thoroughly evaluate the influence of the clinical setting and mortality definitions on the results. Finally, we believe that the publication of non-significant findings might been affected by reporting biases. Studies that found no association between the biomarker values and mortality probably have fewer chances to be published in indexed journals or in other publicly available sources [69]. Besides, we could not analyse the results of several publications due to their incomplete reporting of results, which increases the risk of selective outcome reporting. Researchers and journals in this field are encouraged to publish studies with null results and carefully report properly their results, as this information is crucial for the assessment of the actual value of any biomarker to guide the management of critically ill patients. Our study has not assessed the added predictive value of the biomarkers. Future investigations should specifically evaluate the biomarkers’ added predictive value in the context of other prognostic information.

## Conclusion

The use of resources and related costs for treating sepsis are considerable, so all additional information proposed to guide clinicians in the adequate treatment for each patient need to be supported with the maximum level of evidence to be helpful in the daily clinical setting [2, 8, 70–74]. Biomarkers have the potential to predict or detect sepsis complications, as well as improve prognostication and therefore support clinical management decisions. However, the results of this review showed that unique basal and isolated measurement of PCT, CRP, IL-6, and sCD14 biomarkers are not useful for determining the mortality prognosis among critically ill sepsis patients during their hospital stay. Biomarkers are valuable tools for understanding the biology of sepsis and can guide clinical care by identifying different molecular phenotypes involved in critical illness precision medicine. Before using a biomarker to determine prognosis in clinical practice, we should consider better standardization of clinical studies, which include a group of patients with a specific sepsis phenotype, as well as uniformity in the measurement methods of these biomarkers. Future assessments that integrate these biomarkers with clinical severity scores, individual patient variables, and complex sepsis pathways such as inflammation, clotting or endothelial damage improved patient selection in clinical trials, allowing for the recruitment of more homogenous patient cohorts in objectively predicting adverse outcomes in septic patients [16].

### Supplementary Information

**Additional file 1: Additional file S1.** MEDLINE and EMBASE literature search strategies. **Additional file S2.** QUIPS domains-items considering for assessment. **Additional file S3.** Excluded studies and reason for exclusion. **Additional file S4.** Characteristics of Included Studies. **Additional file S5.** Methodological quality of included studies using the QUIPS tool. **Additional Table S6.** Baseline PCT values and mortality: measure effects. **Additional Table S7.** Baseline CRP values and mortality: measure effects. **Additional Table S8.** Baseline IL-6 values and mortality: measure effects. **Additional Table S9.** Baseline sCD14 values and mortality: measure effects. **Additional Figure S1.** Procalcitonin and prediction of mortality at 28-30 days in critically-ill septic patients. **Additional Figure S2.** C-reactive protein and prediction of mortality at 28-30 days in critically-ill septic patients. **Additional Figure S3.** Interleukin-6 and prediction of mortality at 28-30 days in critically-ill septic patients. **Additional Figure S4.** Summary of mortality at 28-30 days and baseline biomarkers measures. **Additional Figure S5.** Summary of mortality, no details provided and baseline biomarkers measures.

## Data Availability

All data generated or analysed during this study are included in this published article [and its supplementary information files].
