# Supplementary material for: Basal procalcitonin, C-reactive protein, interleukin-6, and presepsin for prediction of mortality in critically ill septic patients: a systematic review and meta-analysis
Source: Diagn Progn Res. 2023 Aug 3;7:15. doi: 10.1186/s41512-023-00152-2 (PMC10399020; doi:10.1186/s41512-023-00152-2)
Supplement: Supplementary file 1 — Additional file 1: Additional file S1. MEDLINE and EMBASE literature search strategies. Additional file S2. QUIPS domains-items considering for assessment. Additional file S3. Excluded studies and reason for exclusion. Additional file S4. Characteristics of Included Studies. Additional file S5. Methodological quality of included studies using the QUIPS tool. Additional Table S6. Baseline PCT values and mortality: measure effects. Additional Table S7. Baseline CRP values and mortality: measure effects. Additional Table S8. Baseline IL-6 values and mortality: measure effects. Additional Table S9. Baseline sCD14 values and mortality: measure effects. Additional Figure S1. Procalcitonin and prediction of mortality at 28-30 days in critically-ill septic patients. Additional Figure S2. C-reactive protein and prediction of mortality at 28-30 days in critically-ill septic patients. Additional Figure S3. Interleukin-6 and prediction of mortality at 28-30 days in critically-ill septic patients. Additional Figure S4. Summary of mortality at 28-30 days and baseline biomarkers measures. Additional Figure S5. Summary of mortality, no details provided and baseline biomarkers measures. [file 41512_2023_152_MOESM1_ESM.docx]

**Additional file S1.** MEDLINE and EMBASE literature search strategies

**Additional file S2.** QUIPS domains-items considering for assessment.

**Additional file S3.** Excluded studies and reason for exclusion

**Additional file S4.** Characteristics of Included Studies

**Additional file S5.** Methodological quality of included studies using the QUIPS tool

**Additional Table S6.** Baseline PCT values and mortality: measure effects

**Additional Table S7.** Baseline CRP values and mortality: measure effects

**Additional Table S8.** Baseline IL-6 values and mortality: measure effects

**Additional Table S9.** Baseline sCD14 values and mortality: measure effects

**Additional Figure S1.** Procalcitonin and prediction of mortality at 28-30 days in critically-ill septic patients

**Additional Figure S2.** C-reactive protein and prediction of mortality at 28-30 days in critically-ill septic patients

**Additional Figure S3.** Interleukin-6 and prediction of mortality at 28-30 days in critically-ill septic patients

**Additional Figure S4.** Summary of mortality at 28-30 days and baseline biomarkers measures

**Additional Figure S5**. Summary of mortality, no details provided and baseline biomarkers measures

**Additional references**

**S1Table.** MEDLINE and EMBASE Literature search strategies

**Ovid MEDLINE(R) ALL <1946 to March 24 2023>**

1 Hemorrhagic Septicemia/

2 exp Sepsis/

3 exp Shock, Septic/

4 exp Critical Illness/

5 exp Bacteremia/

6 Systemic Inflammatory Response Syndrome/

7 (sepsis or septic* or bacter?em*).mp.

8 (Flavimonas oryzihabitans Bacteremia or Systemic Inflammatory Response).mp.

9 (critical* adj3 ill*).ab,ti.

10 1 or 2 or 3 or 4 or 5 or 6 or 7 or 8 or 9

11 exp Interleukin-6/

12 exp Receptors, Interleukin-6/

13 exp Interleukin-6 Receptor alpha Subunit/

14 (interleukin* or IL?6* or IL 6*).mp.

15 11 or 12 or 13 or 14

16 exp Procalcitonin/

17 exp Calcitonin/

18 (procalcitonin or PCT).ab,ti.

19 (calcitonin and precursor*).ab,ti.

20 16 or 17 or 18 or 19

21 exp C-Reactive Protein/

22 (C?Reactive protein* or CRP).ab,ti.

23 21 or 22

24 (presepsin or Solube CD14 or SCD14 or SCD?14).mp.

25 15 or 20 or 23 or 24

26 10 and 25

27 ((child* or nenonat* or infant*) not adult*).af.

28 (animal* not human*).af.

29 26 not 27

30 29 not 28

31 exp COVID-19/

32 exp SARS-CoV-2/

33 (covid-19 or Sars-cov-2).tw.

34 31 or 32 or 33

35 30 not 34

36 (201906* or 201907* or 201908* or 201909* or 201910* or 201911* or 201912* or 2020* or 2021*).ez,tw.

37 35 and 36

**Embase-Elsevier**

#40 #36 NOT #37 AND [embase]/lim AND [17-6-2019]/sd NOT [13-4-2021]/sd

#39 #36 NOT #37 AND [embase]/lim

#38 #36 NOT #37

#37 (child* OR nenonat* OR infant*) NOT adult

#36 #31 NOT #35

#35 #32 OR #33 OR #34

#34 'covid 19' OR 'sars cov 2':ab,ti

#33 'severe acute respiratory syndrome coronavirus 2'/exp

#32 'coronavirus disease 2019'/exp

#31 #29 NOT #30

#30 animal* NOT human*

#29 #10 AND #28

#28 #15 OR #19 OR #22 OR #27

#27 #23 OR #24 OR #25 OR #26

#26 scd?14:ab,kw,ti,de

#25 scd14:ab,kw,ti,de

#24 soluble AND cd14:ab,kw,ti,de

#23 presepsin:ab,ti,kw,de

#22 #20 OR #21

#21 c$reactive AND protein*:ti,ab OR crp:ti,ab

#20 'c reactive protein'/exp

#19 #16 OR #17 OR #18

#18 calcitonin AND precursor:ti,ab

#17 procalcitonin OR pct:ti,ab

#16 'procalcitonin'/exp

#15 #11 OR #12 OR #13 OR #14

#14 interleukin*:ti,ab OR il$6:ti,ab OR ((il NEAR/2 6*):ti,ab)

#13 'interleukin 6 receptor alpha'/exp

#12 'interleukin 6 receptor'/exp

#11'interleukin 6'/exp

#10 #1 OR #2 OR #3 OR #4 OR #5 OR #6 OR #7 OR #8 OR #9

#9 (critical* NEAR/3 ill*):ti,ab

#8 'flavimonas oryzihabitans oryzihabitans bacteremia' OR 'systemic inflammatory response':ti,ab

#7 sepsis OR septic* OR bacter$em*:ti,ab

#6 'systemic inflammatory response syndrome'/exp

#5 'bacteremia'/exp

#4 'critical illness'/exp

#3 'septic shock'/exp

#2 'sepsis'/exp

#1 'hemorrhagic septicemia'/exp

**S2 Table.** QUIPS domains-items considering for assessment.

| **Domain** | **Items considered for assessment** | **Risk of bias rating** |
| --- | --- | --- |
| **1. Study participation** | The source population or population of interest is adequately described for our key characteristics. Baseline characteristics of interest are clearly indicated for the population (e.g., disease severity).  Eligibility criteria and recruitment are adequately described (e.g., diagnosis of sepsis correctly reported). There is a description of the period and place of recruitment.  There is adequate participation in the study by eligible participants. There are no exclusions due to biomarker values or caused by the evolution of the patient. | **LOW**: if all items are indicated as non-problematic, the relationship between the biomarker and outcome is unlikely to be different for participants and eligible nonparticipants.  **MODERATE**: some items are not clearly reported, or some are indicated as problematic.  **HIGH**: if more than one item is reported as problematic, the relationship between the biomarker and outcome is very likely to be different for participants and eligible nonparticipants. |
| **2. Study attrition** | There should be no subsequent unwarranted exclusions or a notable number of non-participants. Furthermore, in the presence of lost follow-ups, there are descriptions of attempts to collect information on participants who dropped out.  In the case of justified exclusions (e.g., no outcome-related or biomarker-related exclusions), there are no important differences among participants who completed the study and those who did not. | **LOW**: if all items are indicated as non-problematic, the relationship between the biomarker and outcome is unlikely to be different for completing and non-completing participants.  **MODERATE**: some items are not clearly reported, or some are indicated as problematic.  **HIGH**: if more than one item is reported as problematic, the relationship between the biomarker and outcome is very likely to be different for completing and non-completing participants. |
| **3. Biomarker measurement** | The laboratory technique or method for measuring the biomarker is clearly reported. The same method and setting of measurement are used in all study participants (e.g., all patients have the same number of samples analysed; in the presence of several biomarkers, there are differences in their measurement).  Methods of measurement were accurate, valid, consistent and reliable, e.g., the information is collected by a laboratory test instead of the medical record.  Continuous variables are treated appropriately, and rationale provided for cut-off values if analysed as categorical. Penalised if the cut-off point is derived in the same sample (e.g., obtaining the cut-off point by ROC).  Adequate proportion of the study sample has complete data for prognostic factors and, if not, appropriate methods of imputation were used for missing data (e.g., group average). | **LOW**: if all items are indicated as non-problematic, the relationship between the biomarker and outcome is unlikely to be different levels of the outcome of interest.  **MODERATE**: some items are not clearly reported, or some are indicated as problematic.  **HIGH**: if more than one item is reported as problematic, the relationship between the biomarker and outcome is very likely to be different levels of the outcome of interest. |
| **4. Mortality measurement** | The assessment of mortality does not require an evaluation of the accuracy of the outcome measure since death is an objectively measurable endpoint. However, the methods of measurement should be accurate, valid, consistent and reliable (e.g., 28-day mortality, ICU mortality, sepsis-related mortality, etc).  The method and setting of measurement were the same for all participants. | **LOW**: if all items are indicated as non-problematic, the measurement of the outcome is unlikely to be different related to the baseline level of the biomarker.  **MODERATE**: some items are not clearly reported, or some are indicated as problematic.  **HIGH**: if more than one item is reported as problematic, the measurement of the outcome is very likely to be different related to the baseline level of the biomarker. |
| **5. Adjustment for other prognostic factors** | All-important confounders are measured, and clear definitions are provided:   - Age (years). - Severity score (SOFA, SAPS-II, APACHE).   The method and setting of measurement were the same for all participants, ensuring accuracy, validity, consistency, and reliability (e.g., validated scales used).  The two important potential confounders are accounted for in the study design or in the analysis, and appropriate methods were used to account for missing data. | **LOW**: if all items are indicated as non-problematic, the observed effect of the biomarker on the outcome is unlikely to be distorted by a confounder.  **MODERATE**: some items are not clearly reported, or some are indicated as problematic.  **HIGH**: if more than one item is reported as problematic, the observed effect of the biomarker on the outcome is very likely to be distorted by a confounder. |
| **6. Statistical analysis and reporting** | There is sufficient presentation of the data to assess the adequacy of the analysis. The strategy for model building was acceptable and based on a conceptual framework (e.g., no model overfitting, 1 variable/10 events). The analysis is appropriate for the design of the study.  There is no selective reporting of results (e.g., measure of effect not only reported when the results are statistically significant or inconsistent reported data). | **LOW**: if all items are indicated as non-problematic, the reported results are unlikely to be spurious or biased related to analysis or reporting.  **MODERATE**: some items are not clearly reported, or some are indicated as problematic.  **HIGH**: if more than one item is reported as problematic, the reported results are very likely to be spurious or biased related to analysis or reporting. |

**S3 Table.** Excluded studies and reason for exclusion

| **ID** | **Reason for exclusion** |
| --- | --- |
| Azevedo 2012 ^1^ | Wrong outcome |
| Calandra 1991 ^2^ | Wrong population |
| Gao C 2022 ^3^ | Wrong prognostic factor |
| Gao Q 2021 ^4^ | Wrong prognostic factor |
| Di 2020 ^5^ | Wrong outcome |
| El Said 2020 ^6^ | Wrong outcome |
| Hashim 2020 ^7^ | Wrong outcome |
| Hillas 2010 ^8^ | Wrong outcome |
| Honorato 2023 ^9^ | Wrong prognostic factor |
| Kandaswamy 2018 ^10^ | Wrong population |
| Kang 2008 ^11^ | Wrong outcome |
| Karampela 2019 ^12^ | Wrong population |
| Kellum 2007 ^13^ | Wrong outcome |
| Kim 2013 ^14^ | Wrong prognostic factor |
| Li Y 2022 ^15^ | Wrong prognostic factor |
| Li Z 2022 ^16^ | Wrong prognostic factor |
| Nanda 2016 ^17^ | Wrong outcome |
| Pieralli 2015 ^18^ | Wrong prognostic factor |
| Schroder 1999 ^19^ | Wrong outcome |
| Schuetz 2007 ^20^ | Wrong prognostic factor |
| Sharma 2014 ^21^ | Wrong study design |
| Stalder 2016 ^22^ | Wrong prognostic factor |
| Su 2012 ^23^ | Wrong outcome |
| Sunden 2005 ^24^ | Wrong outcome |
| Tan 2017 ^25^ | Wrong outcome |
| Thao 2018 ^26^ | Wrong prognostic factor |
| Yao 2016 ^27^ | Wrong prognostic factor |
| Zhang 2019 ^28^ | Wrong prognostic factor |
| Baoquan 2019 ^29^ | Wrong outcome |
| Malek 2019 ^30^ | Wrong study design |
| Naderpour 2019 ^31^ | Wrong study design |
| C. Peng 2020 ^32^ | Wrong prognostic factor |
| Tschaikowsky 2011 ^33^ | Wrong prognostic factor |
| Behnes 2014 ^34^ | Wrong population |
| Frencken 2017 ^35^ | Wrong prognostic factor |
| Goldberg 2021 ^36^ | Wrong prognostic factor |
|  |  |
| Gradel 2013 ^37^ | Wrong population |
| Kurisu 2019 ^38^ | Wrong prognostic factor |
| Matera 2017 ^39^ | Wrong population |
| Owasawara 2020 ^40^ | Wrong prognostic factor |
| Pallas 2016 ^41^ | Wrong population |
| Pauly 2016 ^42^ | Wrong population |
| Peng 2017 ^43^ | Wrong prognostic factor |
| Shen 2020 ^44^ | Wrong population |
| Suarez de la Rica 2015 ^45^ | Wrong prognostic factor |
| Tanriverdi 2015 ^46^ | Wrong prognostic factor |
| Taskin 2016 ^47^ | Wrong prognostic factor |
| Wunder 2004 ^48^ | Wrong prognostic factor |
| Yan 2021 ^49^ | Wrong population |

**S4 Table.** Characteristics of Included Studies

| **Study ID** | **Countries** | **Number of study centers** | **Hospital setting at admission** | **Total sample included in the analysis** | **Age (years)** | **Sex**  **(% females)** | **Criteria used for sepsis definition** | **Sepsis origin** | **APACHE-II** | **SOFA** | **SAPS II** | **PCT** | **CRP** | **IL6** | **PSP, SCD14** |
| --- | --- | --- | --- | --- | --- | --- | --- | --- | --- | --- | --- | --- | --- | --- | --- |
| Aalto 2007 | Finland | Single center | Emergency room | 142 | 60  Expressed as  **Mean** | 59 (41.5%) | Mix | Mixed | NR | NR | NR | No | Yes | No | Yes |
| Amancio 2013 | Brazil | Multi-center – 4 centers | ICU | 60 | 32 (26; 43)  66 (46; 78)  Expressed as  **Median (25th – 75th)** | 20 (33.0%) | ACCP/SCCM 1991 criteria | Mixed | NR | 8 (6; 10.5)  10 (9; 13)  Expressed as  **Median** | 55 (47; 58)  55 (44; 63)  Expressed as  **Median** | No | Yes | Yes | No |
| Andaluz-Ojeda 2012 | Spain | Single center | ICU | 29 | 66.1  Expressed as  **Mean** | 12 (41.3%) | Levy criteria (2001 SCCM/ESICM/ACCP/ATS/SIS International Sepsis Definitions Conference) | Mixed | 22.2  Expressed as  **Mean** | 8.1  Expressed as  **Mean** | NR | No | No | Yes | No |
| Andaluz-Ojeda 2017 | Spain-France | Multi-center – 2 centers | ICU | 326 | 65.4 (14)  Expressed as  **Mean (SD)** | 105 (38.6%) | Levy criteria (2001 SCCM/ESICM/ACCP/ATS/SIS International Sepsis Definitions Conference) | Mixed | NR | 9 (3.7)  Expressed as  **Mean** | NR | Yes | Yes | No | No |
| Belli 2022 | Italy | Single center | ICU | 35 | 59 (48; 60)  Expressed as  **Median (25th – 75th)** | 14 (40.0%) | Singer criteria (2015 SCCM/ESICM Sepsis-3) | Mixed | NR | NR | NR | Yes | No | Yes | No |
| Carbonell 2021 | Spain | Multi-center – more than 5 centers | ICU | 422 | 59 (49; 71)  61 (48; 74)  Expressed as  **Median (25th – 75th)** | 159 (37.7%) | Levy criteria (2001 SCCM/ESICM/ACCP/ATS/SIS International Sepsis Definitions Conference) | Respiratory | 18 (13; 24)  23 (18; 28)  Expressed as  **Median (25th – 75th)** | 6 (4; 9)  9 (6.2; 12)  Expressed as  **Median (25th – 75th)** | NR | Yes | Yes | No | No |
| Chen 2020 | China | Single center | ICU | 120 | 50.3 (8.4)  Expressed as  **Mean (SD)** | 66 (55.0%) | Singer criteria (2015 SCCM/ESICM Sepsis-3) | Mixed | 15.2 (4.6)  Expressed as  **Mean (SD)** | 7.3 (3.8)  Expressed as  **Mean (SD)** | NR | Yes | No | No | No |
| De la Torre-Prados 2016 | Spain | Single center | ICU | 100 | 64 (56; 72)  Expressed as  **Median (25th – 75th)** | 41 (41.0%) | Levy criteria (2001 SCCM/ESICM/ACCP/ATS/SIS International Sepsis Definitions Conference) | Mixed | 26 (8)  Expressed as  **Median (IQR)** | 10 (4)  Expressed as  **Median (IQR)** | NR | Yes | Yes | No | No |
| Eidt 2016 | Brazil | Single center | ICU | 48 | 61.4 (18.7)  65.9 (17.8)  Expressed as  **Mean (SD)** | 24 (50.0%) | Levy criteria (2001 SCCM/ESICM/ACCP/ATS/SIS International Sepsis Definitions Conference) | NR | 11.4 (4.7)  16.0 (6.7)  Expressed as  **Mean (SD)** | 7.2 (3.0)  7.6 (5.2)  Expressed as  **Mean (SD)** | NR | No | No | Yes | No |
| Elke 2018 | Germany | Multi-center – more than 5 centers | ICU | 1076 | 65.7 (13.7)  Expressed as  **Mean (SD)** | 395 (36.7%) | Singer criteria (2015 SCCM/ESICM Sepsis-3) | Mixed | 24.2 (7.6)  Expressed as  **Mean (SD)** | 10.3 (3.3)  Expressed as  **Mean (SD)** | 63.2 (14.1)  Expressed as  **Mean (SD)** | Yes | Yes | No | No |
| Erdogan 2021 | Turkey | Single center | ICU | 187 | 74 (66; 80)  Expressed as  **Median (25th – 75th)** | 105 (56.0%) | Singer criteria (2015 SCCM/ESICM Sepsis-3) | NR | 19 (16; 24)  33 (26; 39)  Expressed as  **Median (25th – 75th)** | 5 (5; 8)  6 (6; 12)  Expressed as  **Median (25th – 75th)** | NR | Yes | Yes | No | No |
| Gao C 2022 | China | Single center | ICU | 205 | 45 (36; 54)  Expressed as  **Median (25th – 75th)** | 84 (40.9%) | Other | NR | 16.4 (5.6)  Expressed as  **Mean (SD)** | 9.8 (3.4)  Expressed as  **Mean (SD)** | NR | Yes | No | No | No |
| Guo 2018 | China | Single center | ICU | 59 | 63.6 (17.9)  Expressed as  **Mean (SD)** | 17 (28.8%) | singer criteria (2015 SCCM/ESICM Sepsis-3) | Mixed | 15 (9; 21)  Expressed as  **Median (25th – 75th)** | 4 (3; 8)  Expressed as  **Median (25th – 75th)** | NR | Yes | Yes | No | No |
| Hu 2018 | China | Single center | ICU | 141 | 63 (33; 78)  Expressed as  **Median (25th – 75th)** | 55 (39.0%) | Singer criteria (2015 SCCM/ESICM Sepsis-3) | Mixed | 21 (10; 36)  Expressed as  **Median (25th – 75th)** | 5 (2; 14)  Expressed as  **Median (25th – 75th)** | NR | Yes | Yes | No | No |
| Huang N 2022 | China | Single center | ICU | 349 | 66 (52; 77)  Expressed as  **Median (25th – 75th)** | 120(34.4%) | Singer criteria (2015 SCCM/ESICM Sepsis-3) | Mixed | 20 (14.5; 26)  Expressed as  **Median (25th – 75th)** | 8 (5; 11)  Expressed as  **Median (25th – 75th)** | NR | Yes | Yes | No | No |
| Jain 2014 | India | Single center | ICU | 54 | 50.7 (18.7)  Expressed as  **Mean (SD** | 24 (46.0%) | Levy criteria (2001 SCCM/ESICM/ACCP/ATS/SIS International Sepsis Definitions Conference) | Mixed | 24.7 (7.8)  Expressed as  **Mean (SD)** | 8.3 (3.7)  Expressed as  **Mean (SD)** | 53.2 (16.8)  Expressed as  **Mean (SD)** | Yes | Yes | No | No |
| Jekarl 2019 | South Korea | Single center | Emergency room | 185 | 70.1 (14.9)  Expressed as  **Mean (SD)** | 124 (67.0%) | Singer criteria (2015 SCCM/ESICM Sepsis-3) | Mixed | NR | 3.54 (2.81)  4.65 (3.12)  Expressed as  **Mean (SD)** | NR | Yes | Yes | No | No |
| Jiang 2019 | China | Single center | ICU | 198 | 69.9 (29; 91)  68.7 (18; 91)  Expressed as  **Median (25th – 75th)** | 72 (18.2%) | Singer criteria (2015 SCCM/ESICM Sepsis-3) | Mixed | NR | 7 (4; 13)  10 (6; 15)  Expressed as  **Median (25th – 75th)** | NR | No | No | Yes | No |
| Karamouzos 2021 | Greece | NR | ICU | 128 | 72.4 (15)  77.3 (10)  Expressed as  **Mean (SD)** | 72 (56.2%) | Levy criteria (2001 SCCM/ESICM/ACCP/ATS/SIS International Sepsis Definitions Conference) | Mixed | 19 (6.6)  22 (6.3)  Expressed as  **Mean (SD)** | 6.5 (4)  9.2 (3.6)  Expressed as  **Mean (SD)** | NR | No | No | Yes | No |
| Karampela 2022 | Greece | Single center | ICU | 102 | 64.7 (15.6)  Expressed as  **Mean (SD)** | 45 (44.1%) | Singer criteria (2015 SCCM/ESICM Sepsis-3) | Mixed | 23 (7.2)  Expressed as  **Mean (SD)** | 10 (3.3)  Expressed as  **Mean (SD)** | NR | No | No | Yes | No |
| Khashaba 2022 | Egypt | Single center | ICU | 178 | 53.9 (10.3)  57.4 (8.6)  Expressed as  **Mean (SD)** | 71 (39.9%) | Singer criteria (2015 SCCM/ESICM Sepsis-3) | NR | 22.5 (4.1)  24.4 (6.8)  Expressed as  **Mean (SD)** | 9.5 (2.5)  10.5 (2.7)  Expressed as  **Mean (SD)** | NR | Yes | No | No | Yes |
| Kim S 2022 | Korea | Single center | ICU | 282 | 60.6  Expressed as  **Mean** | 80 (28.4%) | Other | Respiratory | 25.3  Expressed as  **Mean (SD)** | NR | NR | No | Yes | No | No |
| Koozi 2023 | Sweden | Multi-center – 4 centers | ICU | 1984 | 69 (59; 76)  Expressed as  **Median (25th – 75th)** | 831 (41.9%) | Singer criteria (2015 SCCM/ESICM Sepsis-3) | NR | NR | 7 (5; 10)  Expressed as  **Median (25th – 75th)** | NR | No | Yes | No | No |
| Lai X 2022 | China | Single center | ICU | 117 | 56 (52; 59)  59 (46; 71)  Expressed as  **Median (25th – 75th)** | 58 (49.6%) | Singer criteria (2015 SCCM/ESICM Sepsis-3) | Mixed | 22(16; 32)  18(13; 23)  Expressed as  **Median (25th – 75th)** | 10.5 (7.3; 14.5)  6.9 (3.7; 9.9)  Expressed as  **Median (25th – 75th)** | NR | Yes | No | Yes | No |
| Lee J 2021 | Korea | Single center | ICU | 100 | 71.0 (61.0; 79.0)  69.5 (54.0; 83.0)  Expressed as  **Median (25th – 75th)** | 41 (46.6%) | Singer criteria (2015 SCCM/ESICM Sepsis-3) | NR | NR | 8.0 (7.0; 11.0)  11.0 (8.0; 13.0)  Expressed as  **Median (25th – 75th)** | 72.2 (11.6)  83.6 (12.9)  Expressed as  **Median (25th – 75th)** | Yes | No | No | Yes |
| Lee S 2022 | Korea | Single center | Emergency room | 278 | 77 (64; 84)  Expressed as  **Median (25th – 75th)** | 116 (42.0%) | Singer criteria (2015 SCCM/ESICM Sepsis-3) | Mixed | 26 (22; 32)  29 (25; 33)  Expressed as  **Median (25th – 75th)** | 6 (5; 8)  10 (8; 12)  Expressed as  **Median (25th – 75th)** | NR | No | No | No | Yes |
| Li 2019 | China | Single center | ICU | 245 | 84.3 (7.6)  Expressed as  **Mean (SD)** | 58 (23.7%) | NR | Mixed | 13.8 (5.1)  Expressed as  **Mean (SD)** | NR | NR | No | Yes | No | No |
| Li X 2021 Clinica | China | Single center | ICU | 220 | 69 (11)  63 (18)  Expressed as  **Mean (SD)** | 68 (30.9%) | Singer criteria (2015 SCCM/ESICM Sepsis-3) | Mixed | NR | 11 (4:21)  5 (2;15)  Expressed as  **Median (25th – 75th)** | NR | Yes | No | No | No |
| Liu 2021 | China | Single center | ICU | 264 | 52.9 (12.6)  Expressed as  **Mean (SD)** | 97 (36.0%) | Singer criteria (2015 SCCM/ESICM Sepsis-3) | Mixed | 20.79 (6.89)  Expressed as  **Mean (SD)** | 8 (6; 11)  Expressed as  **Median (25th – 75th)** | NR | Yes | Yes | Yes | No |
| Masson 2014 | Italy | Multi-center – more than 5 centers | ICU | 100 | 71.6 (10.8)  71.3 (13.6)  Expressed as  **Mean (SD)** | 46 (46.0%) | Mix | Mixed | NR | 8 (6; 10)  9 (7; 11)  Expressed as  **Median (25th – 75th)** | 50 (14)  51 (12)  Expressed as  **Mean (SD)** | Yes | No | No | Yes |
| Oberholzer 2015 | USA | Multi-center – more than 5 centers | Mixed | 124 | 58.3 (17.5)  Expressed as  **Mean (SD)** | 56 (45.0%) | Other | NR | 21.6 (8.2)  Expressed as  **Mean (SD)** | NR | NR | Yes | No | Yes | No |
| Phua 2008 | Singapore | Single center | ICU | 72 | 55 (16)  54 (17)  Expressed as  **Mean (SD)** | 26 (36.0%) | Levy criteria (2001 SCCM/ESICM/ACCP/ATS/SIS International Sepsis Definitions Conference) | Mixed | 23.1 (7.5)  32.3 (8.7)  Expressed as  **Mean (SD)** | 10.1 (3.0)  12.7 (4.4)  Expressed as  **Mean (SD)** | NR | Yes | No | Yes | No |
| Ryoo 2019 | South Korea | Multi-center – more than 5 centers | Emergency room | 1772 | 67.5 (13.6)  Expressed as  **Mean (SD)** | 727 (41.0%) | NR | Mixed | 19 (14; 26)  Expressed as  **Median (25th – 75th)** | 8 (5; 11)  Expressed as  **Median (25th – 75th)** | NR | Yes | Yes | No | No |
| Shao 2015 | China | Single center | Emergency room | 227 | 71 (66; 78)  73 (62; 77)  74 (64; 79)  Expressed as  **Median (25th – 75th)** | 88 (38.7%) | Levy criteria (2001 SCCM/ESICM/ACCP/ATS/SIS International Sepsis Definitions Conference) | Mixed | NR | NR | NR | Yes | No | No | No |
| Shimoyama 2021 | Japan | Single center | ICU | 83 | 74.0 (65.5; 78.5)  Expressed as  **Median (25th – 75th)** | 32 (38.6%) | Singer criteria (2015 SCCM/ESICM Sepsis-3) | NR | NR | 8.0 (5; 11)  Expressed as  **Median (25th – 75th)** | NR | No | No | No | Yes |
| Siddiqui 2019 | Singapore | Single center | ICU | 198 | 63.0 (49.5; 73.5)  54.0 (45.0; 64.0)  61.0 (40.0; 68.8)  Expressed as  **Mean (SD)** | 78 (39.4%) | NR | NR | NR | NR | NR | No | No | Yes | No |
| Song 2019 | South Korea | Single center | Emergency room | 97 | 75 (42; 98)  Expressed as  **Median (25th – 75th)** | 43 (44.3%) | Singer criteria (2015 SCCM/ESICM Sepsis-3) | Mixed | 21 (13; 30)  Expressed as  **Median (25th – 75th)** | 8 (4; 11)  Expressed as  **Median (25th – 75th)** | NR | Yes | Yes | Yes | No |
| Su 2013 | China | Single center | ICU | 100 | 58.9 (19.5)  Expressed as  **Mean (SD)** | 33 (33.0%) | Levy criteria (2001 SCCM/ESICM/ACCP/ATS/SIS International Sepsis Definitions Conference) | Mixed | 13.4 (6.1)  Expressed as  **Mean (SD)** | 7.8 (4.4)  Expressed as  **Mean (SD)** | NR | Yes | Yes | No | No |
| Suberviola 2013 | Spain | Single center | ICU | 137 | 62.6 (15.9)  Expressed as  **Mean (SD)** | 47 (34.3%) | Levy criteria (2001 SCCM/ESICM/ACCP/ATS/SIS International Sepsis Definitions Conference) | Mixed | 20.6 (7.2)  Expressed as  **Mean (SD)** | 8.6 (3.0)  Expressed as  **Mean (SD)** | NR | Yes | Yes | No | No |
| Sun 2011 | china | Single center | ICU | 50 | 66 (17)  Expressed as  **Mean (SD)** | 20 (40.0%) | ACCP/SCCM 1991 criteria | NR | 19.0 (5.8)  24.9 (6.0)  Expressed as  **Mean (SD)** | NR | NR | Yes | Yes | No | No |
| Suranadi 2022 | Indonesia | Single center | ICU | 228 | 53.6 (16.4)  Expressed as  **Mean (SD)** | 94 (41.2%) | Levy criteria (2001 SCCM/ESICM/ACCP/ATS/SIS International Sepsis Definitions Conference) | Mixed | NR | 7.1 (3.25)  Expressed as  **Mean (SD)** | NR | Yes | No | No | No |
| Taha 2023 | Egypt | Single center | ICU | 54 | 54.4 (9.3)  40.5 (9.6)  Expressed as  **Mean (SD)** | 23 (42.6%) | Singer criteria (2015 SCCM/ESICM Sepsis-3) | Mixed | NR | 29.33 (5.28  29.72 (5.85  Expressed as  **Mean (SD)** | NR | No | Yes | No | No |
| Tian 2021 | China | Single center | ICU | 194 | 78 (63.5; 83)  81 (76; 86.5)  Expressed as  **Median (25th – 75th)** | 76 (38.2%) | Singer criteria (2015 SCCM/ESICM Sepsis-3) | Mixed | 18.0 (13.0; 26.3)  36.0 (32.0; 39.0)  Expressed as  **Median (25th – 75th)** | 4.0 (3.0; 6.0)  5.5 (4.0; 9.0)  Expressed as  **Median (25th – 75th)** | NR | Yes | Yes | No | No |
| Tokur 2022 | Turkey | Single center | ICU | 39 | 72 (52; 84)  Expressed as  **Median (25th – 75th)** | 17 (43.6%) | Other | Bacteriemia | NR | NR | 63 (58; 70)  41 (25; 51.7)  Expressed as  **Median (25th – 75th)** | No | Yes | No | No |
| Viallon 2008 | France | Single center | Mixed | 131 | 71 (15)  74 (14)  61 (21)  Expressed as  **Mean (SD)** | NR | Levy criteria (2001 SCCM/ESICM/ACCP/ATS/SIS International Sepsis Definitions Conference) | Mixed | NR | NR | 22.2 (1.0)  50.2 (3.6)  Expressed as  **Mean (SD)** | Yes | Yes | Yes | No |
| Wang 2014 | China | Single center | Emergency room | 360 | 71 (59; 78)  73 (60; 78)  73 (65; 78)  Expressed as  **Median (25th – 75th)** | 178 (40.4%) | ACCP/SCCM 1991 criteria | Mixed | NR | NR | NR | Yes | No | No | No |
| Webb 2020 | USA | Single center | Emergency room | 148 | 72 (60; 82)  Expressed as  **Median (25th – 75th)** | 67 (45.3%) | Singer criteria (2015 SCCM/ESICM Sepsis-3) | Mixed | NR | NR | NR | yes | No | No | No |
| Wu CX 2021 | China | Single center | ICU | 114 | 72 (60; 81)  Expressed as  **Median (25th – 75th)** | 31 (27.1%) | Singer criteria (2015 SCCM/ESICM Sepsis-3) | Mixed | 17.5 (12.75; 22)  Expressed as  **Median (25th – 75th)** | 7 (4; 11)  Expressed as  **Median (25th – 75th)** | NR | Yes | Yes | Yes | No |
| Wu HP 2009 | China | Single center | Mixed | 63 | 70.0 (2.0)  69.1 (4.1)  Expressed as  **Mean (SD)** | 23 (36.5%) | Other | Respiratory | 22.7 (1.0)  28.7 (2.3)  Expressed as  **Mean (SD)** | NR | NR | No | No | Yes | No |
| Xia 2020 | China | Single center | Emergency room | 821 | 56.8 (17.8)  59.9 (17.6)  Expressed as  **Mean (SD)** | 293 (35.6%) | Levy criteria (2001 SCCM/ESICM/ACCP/ATS/SIS International Sepsis Definitions Conference) | Mixed | 15.2 (7.2)  20.5 (7.1)  Expressed as  **Mean (SD)** | 4.7 (3.2)  9.1 (5.0)  Expressed as  **Mean (SD)** | NR | Yes | No | No | No |
| Xie 2021 | China | Multi-center – 2 centers | Emergency room | 90 | 74 (26; 97)  Expressed as  **Median (25th – 75th)** | 32 (35.0%) | Singer criteria (2015 SCCM/ESICM Sepsis-3) | Mixed | NR | 7.5 (4.3)  Expressed as  **Mean (SD)** | NR | Yes | Yes | Yes | No |
| Xie 2023 | China | Single center | Emergency room | 367 | 73 (19; 98)  Expressed as  **Median (25th – 75th)** | 115 (34.1%) | Singer criteria (2015 SCCM/ESICM Sepsis-3) | Mixed | NR | NR | NR | Yes | No | Yes | No |
| Yang 2019 | China | Single center | ICU | 185 | 68.3 (17.1)  Expressed as  **Mean (SD)** | 71 (38.3%) | Other | NR | 24.1 (5.9)  21.6 (6.0)  Expressed as  **Mean (SD)** | 2.7 (0.5)  2.3 (0.6)  Expressed as  **Mean (SD)** | NR | Yes | No | No | No |
| Yin 2013 | China | Single center | Emergency room | 680 | 72 (60; 78)  Expressed as  **Median (25th – 75th)** | 264 (38.8%) | Levy criteria (2001 SCCM/ESICM/ACCP/ATS/SIS International Sepsis Definitions Conference) | Mixed | NR | NR | NR | Yes | No | No | No |
| Yu 2022 | China | Single center | Emergency room | 63 | 79 (34; 95)  Expressed as  **Median (25th – 75th)** | 23 (36.5%) | Singer criteria (2015 SCCM/ESICM Sepsis-3) | NR | 3.56 (11.91)  Expressed as  **Mean (SD)** | 5 (3; 6)  Expressed as  **Median (25th – 75th)** | NR | No | No | Yes | No |
| Zhang 2019 | China | Single center | ICU | 150 | 70 (24; 91)  74.5 (24; 89)  Expressed as  **Median (25th – 75th)** | 55 (36.6%) | Singer criteria (2015 SCCM/ESICM Sepsis-3) | Mixed | NR | 4 (2; 10)  6 (4; 8)  Expressed as  **Median (25th – 75th)** | NR | Yes | No | Yes | No |
| Zhang J 2021 | China | Single center | Emergency room | 236 | 76 (59; 88)  Expressed as  **Median (25th – 75th)** | 98 (41.5%) | Singer criteria (2015 SCCM/ESICM Sepsis-3) | Mixed | 22.5 (5.2)  Expressed as  **Mean (SD)** | NR | NR | Yes | Yes | No | No |
| Zhang LT 2022 | China | Single center | ICU | 75 | 71.5 (14.7)  75.7 (13.4)  Expressed as  **Mean (SD)** | 21 (28.0%) | Singer criteria (2015 SCCM/ESICM Sepsis-3) | Mixed | 18 (15; 23)  0.5 (16.5; 25.5)  Expressed as  **Median (25th – 75th)** | NR | NR | No | No | Yes | No |
| Zhao 2013 | China | Single center | Emergency room | 501 | 74 (61; 80)  Expressed as  **Median (25th – 75th)** | 222 (44.3%) | Levy criteria (2001 SCCM/ESICM/ACCP/ATS/SIS International Sepsis Definitions Conference) | NR | NR | NR | NR | Yes | Yes | Yes | No |
| Zhao J 2020 | China | Multi-center – 2 centers | Emergency room | 316 | 63.8 (0.9)  Expressed as  **Mean (SD)** | 115 (36.4%) | Singer criteria (2015 SCCM/ESICM Sepsis-3) | Mixed | NR | 6 (4; 9)  Expressed as  **Median (25th – 75th)** | NR | No | Yes | No | No |
| Zhao M 2020 | China | Single center | ICU | 478 | 60.5 (12.3)  Expressed as  **Mean (SD)** | 166 (34.7%) | Singer criteria (2015 SCCM/ESICM Sepsis-3) | Mixed | 24.6 (7.0)  Expressed as  **Mean (SD)** | 7.8 (3.3)  Expressed as  **Mean (SD)** | NR | Yes | No | No | No |

**S5 Table.** Methodological quality of included studies using the QUIPS tool

**PCT assessments**

| **Study** | **Outcome** | **Outcome category** | **D1-Study participation: risk of bias rating (high/low/moderate)** | **D2-Study attrition: risk of bias rating (high/low/moderate)** | **D3-Biomarker measurement: risk of bias rating (high/low/moderate)** | **D4-Outcome measurement: risk of bias rating (high/low/moderate)** | **D5-Adjustment for other prognostic factors: risk of bias rating (high/low/moderate)** | **D6-Statistical analysis and reporting: risk of bias rating (high/low/moderate)** | **Model adjusted by** |
| --- | --- | --- | --- | --- | --- | --- | --- | --- | --- |
| Andaluz-Ojeda 2017 | Hospital mortality during the first 28-30 days | Mortality/general at 28-30 days | MODERATE | HIGH | MODERATE | MODERATE | MODERATE | MODERATE | Age, septic shock, cardiovas-cular disease, immunosuppression, chronic renal failure, neoplasia, respiratory source of infection, renal replace-ment therapy, hospital (Valladolid/Dijon), presence of fungal infection, limitation of therapeutic effort. |
| Carbonell 2021 | ICU mortality (no-survival), no details provided | Mortality/general, no details | HIGH | HIGH | LOW | MODERATE | MODERATE | MODERATE | SOFA, chronic heart failure, hematological disease, acute renal failure, mechanical ventilation, shock |
| Chen 2020 | Hospital mortality during the first 28-30 days | Mortality/general at 28-30 days | MODERATE | HIGH | MODERATE | MODERATE | MODERATE | HIGH | Septic shock, MALAT1, Lac, SOFA, APACHE II |
| De la Torre Prado 2016 | Mortality during the first 28-30 days, no details provided | Mortality/general at 28-30 days | LOW | MODERATE | LOW | MODERATE | MODERATE | MODERATE | MR-proADM, APACHE II, SOFA, PCT |
| Elke 2018 | Mortality during the first 28-30 days, no details provided | Mortality/general at 28-30 days | LOW | HIGH | MODERATE | MODERATE | MODERATE | HIGH | Age, presence of comorbidities and septic shock, lactate, SOFA, APACHE-II, SAPS-II |
| Elke 2018 | ICU mortality (no-survival), no details provided | Mortality/general, no details | LOW | HIGH | MODERATE | MODERATE | MODERATE | HIGH | Age, presence of comorbidities and septic shock, lactate, SOFA, APACHE-II, SAPS-II |
| Elke 2018 | Hospital mortality (no-survival), no details provided | Mortality/general, no details | LOW | HIGH | MODERATE | MODERATE | MODERATE | HIGH | Age, presence of comorbidities and septic shock, lactate, SOFA, APACHE-II, SAPS-II |
| Erdogan 2021 | Mortality during the first 28-30 days, no details provided | Mortality/general at 28-30 days | LOW | MODERATE | MODERATE | MODERATE | MODERATE | HIGH | Not adjusted |
| Guo 2018 | Mortality during the first 28-30 days, no details provided | Mortality/general at 28-30 days | HIGH | MODERATE | LOW | MODERATE | HIGH | HIGH | arterial lactate and NT-proBNP |
| Hu 2018 | All-cause mortality at 28-days | Mortality/general at 28-30 days | MODERATE | MODERATE | MODERATE | MODERATE | HIGH | HIGH | Septic shock, SOFA score, APACHE II, Lactate and PTX3 |
| Jain 2014 | Sepsis-related hospital mortality measured at 28-30 days | Mortality/general at 28-30 days | LOW | LOW | MODERATE | MODERATE | MODERATE | HIGH | Renal dysfunction, low mean blood pressure,APACHE II, SOFA, SAPS II score, serum lactate |
| Jekarl 2019 | In-hospital mortality, no details | Mortality/general, no details | LOW | MODERATE | MODERATE | LOW | LOW | MODERATE | Age, WBC, ESR and SOFA score |
| Liu 2021 | Mortality during the first 28-30 days, no details provided | Mortality/general at 28-30 days | MODERATE | LOW | low | MODERATE | LOW | LOW | Age, sex, BMI, SBP, APACHE II, and SOFA scores. |
| Masson 2014 | Mortality during the first 28-30 days | Mortality/general at 28-30 days | LOW | MODERATE | LOW | MODERATE | LOW | MODERATE | SAPS II score, SOFA score, Serum lactate concentratio, mean arterial pressure, central venous oxygen saturation and randomized treatment (matched by age and sex) |
| Masson 2014 | ICU mortality, no details provided | Mortality/general, no details | LOW | MODERATE | LOW | MODERATE | LOW | MODERATE | SAPS II score, SOFA score, Serum lactate concentratio, mean arterial pressure, central venous oxygen saturation and randomized treatment (matched by age and sex) |
| Oberholzer 2005 | Mortality measured at 28-30 days | Mortality/general at 28-30 days | HIGH | MODERATE | LOW | LOW | LOW | HIGH | Not adjusted |
| Phua 2008 | Mortality measured at 28-30 days | Mortality/general at 28-30 days | LOW | LOW | LOW | MODERATE | MODERATE | HIGH | Not adjusted |
| Ryoo 2019 | Mortality measured at 28-30 days | Mortality/general at 28-30 days | HIGH | MODERATE | MODERATE | LOW | LOW | HIGH | Age, pneumonia, UTI, SOFA, APACHE-II, creatinine, lactate level |
| Shao 2015 | Mortality at 28-days | Mortality/general at 28-30 days | LOW | MODERATE | MODERATE | MODERATE | MODERATE | MODERATE | MEDS score, ALC, BTLA+/CD4+T cells, MFI of BTLA |
| Song 2019 | Mortality measured at 28-30 days | Mortality/general at 28-30 days | MODERATE | MODERATE | MODERATE | MODERATE | MODERATE | HIGH | Not adjusted |
| Su 2013 | Mortality measured at 28-30 days | Mortality/general at 28-30 days | LOW | MODERATE | MODERATE | MODERATE | LOW | HIGH | Sex, age, temperature, serum sTREM-1, WBC, APACHE-II, SOFA, use of life support technology, etiological factors, pathogens |
| Suberviola 2013 | In-hospital mortality, no details | Mortality/general, no details | LOW | MODERATE | MODERATE | MODERATE | LOW | HIGH | Sex, age, immunosupression status, APACHE-II |
| Viallon 2008 | Mortality measured at 28-30 days | Mortality/general at 28-30 days | MODERATE | MODERATE | MODERATE | MODERATE | MODERATE | HIGH |  |
| Wang 2014 | Mortality measured at 28-30 days | Mortality/general at 28-30 days | MODERATE | MODERATE | MODERATE | MODERATE | MODERATE | HIGH | NGAL, MMP-9, TIMP-1, MEDS score |
| Webb 2020 | In-hospital mortality, no details | Mortality/general, no details | MODERATE | MODERATE | MODERATE | MODERATE | HIGH | HIGH | Age, gender |
| Xia 2020 | Sepsis-related mortality measured at 28-30 days | Mortality/general at 28-30 days | HIGH | LOW | MODERATE | MODERATE | HIGH | HIGH | Fibrinogen, Lactic acid, albumin, oxygenation index |
| Xie 2021 | Sepsis-related mortality measured at 28-30 days | Mortality/general at 28-30 days | LOW | LOW | MODERATE | MODERATE | HIGH | HIGH | Neutrophil-to-WBC, lactate |
| Yang 2019 | Mortality during the first 28-30 days, no details provided | Mortality/general at 28-30 days | LOW | MODERATE | MODERATE | MODERATE | LOW | MODERATE | Age, sex, GCS, qSOFA; APACHE II, MODS, Myo, cTnl, Lactate |
| Yin 2013 | All-cause mortality during the first 28-30 days | Mortality/general at 28-30 days | LOW | MODERATE | MODERATE | LOW | LOW | MODERATE | Age, sex, types of infection, comorbidities, sTM, D-Dimer, PCT and the MEDS score |
| Zhang 2019 | 28 day mortality | Mortality/general at 28-30 days | HIGH | MODERATE | MODERATE | MODERATE | MODERATE | HIGH | SOFA, NT-proBNP, IL-6, Prothombin time, thrombin time |
| Zhao 2013 | 28 day mortality | Mortality/general at 28-30 days | LOW | LOW | LOW | LOW | LOW | LOW | MEDS score and age |
| Zhao M 2020 | Mortality during the first 28-30 days, no details provided | Mortality/general at 28-30 days | LOW | MODERATE | MODERATE | MODERATE | MODERATE | MODERATE | SOFA, APACHE-II, CVP, PAO/FIO2, ALT, SCr, LACTATE, 24H LCR |
| Belli 2022 | 30 day mortality | Mortality/general at 28-30 days | LOW | MODERATE | MODERATE | MODERATE | HIGH | HIGH | Univariable |
| Gao 2022 | 28 day mortality | Mortality/general at 28-30 days | LOW | MODERATE | MODERATE | MODERATE | LOW | LOW | Age + Sofa+APACHE+ PCT + mir 127 |
| Huang 2022 | 28 day mortality | Mortality/general at 28-30 days | MODERATE | MODERATE | LOW | LOW | HIGH | HIGH |  |
| Karampela 2022 | 28-day mortality | Mortality/general at 28-30 days | HIGH | MODERATE | LOW | MODERATE | MODERATE | HIGH | Cheperin APACHE IL6 |
| Khashab 2022 | 28-day mortality | Mortality/general at 28-30 days | LOW | MODERATE | LOW | MODERATE | MODERATE | HIGH | SOFA, NEWS2 PSP |
| Lai 2022 | 28-day mortality | Mortality/general at 28-30 days | LOW | LOW | LOW | MODERATE | MODERATE | HIGH | HE4, SOFA, IL-6 |
| Lee J 2021 | in-hospital mortality, no details | Mortality/general, no details | HIGH | MODERATE | MODERATE | MODERATE | HIGH | HIGH |  |
| Li X 2021 | 28-day mortality | Mortality/general at 28-30 days | LOW | MODERATE | MODERATE | MODERATE | HIGH | HIGH |  |
| Suranadi 2022 | 28-day mortality | Mortality/general at 28-30 days | MODERATE | HIGH | LOW | LOW |  | MODERATE | Age, Sex CCI Tipo de UCI, Uso de antibiotico, Microorganismo |
| Taha 2023 | 28-day mortality | Mortality/general at 28-30 days | LOW | LOW | LOW | MODERATE | HIGH | HIGH | MPV, PLT, PDW |
| Xie 2023 | 28-day mortality | Mortality/general at 28-30 days | LOW | MODERATE | LOW | MODERATE | HIGH | HIGH | IL-6 PCT dia 1 y dia 3 Lactato |
| Zhang 2021 | 28-day mortality | Mortality/general at 28-30 days | LOW | LOW | MODERATE | MODERATE | HIGH | HIGH | Lactato RDW Shocjk septico |

**CRP assessments**

| **Study** | **Outcome** | **Outcome category** | **D1-Study participation: risk of bias rating (high/low/moderate)** | **D2-Study attrition: risk of bias rating (high/low/moderate)** | **D3-Biomarker measurement: risk of bias rating (high/low/moderate)** | **D4-Outcome measurement: risk of bias rating (high/low/moderate)** | **D5-Adjustment for other prognostic factors: risk of bias rating (high/low/moderate)** | **D6-Statistical analysis and reporting: risk of bias rating (high/low/moderate)** | **Model adjusted by** |
| --- | --- | --- | --- | --- | --- | --- | --- | --- | --- |
| Andaluz-Ojeda 2017 | Hospital mortality during the first 28-30 days | Mortality/general at 28-30 days | MODERATE | HIGH | MODERATE | MODERATE | MODERATE | HIGH | Not adjusted |
| Carbonell 2021 | ICU mortality (no-survival), no details provided | Mortality/general, no details | HIGH | HIGH | LOW | MODERATE | MODERATE | HIGH | SOFA, chronic heart failure, hematological disease, acute renal failure, mechanical ventilation, shock |
| De la Torre Prado 2016 | Mortality during the first 28-30 days, no details provided | Mortality/general at 28-30 days | LOW | MODERATE | LOW | MODERATE | MODERATE | MODERATE | MR-proADM, APACHE II, SOFA, CRP |
| Elke 2018 | Mortality during the first 28-30 days, no details provided | Mortality/general at 28-30 days | LOW | HIGH | MODERATE | MODERATE | MODERATE | HIGH | Age, presence of comorbidities and septic shock, lactate, SOFA, APACHE-II, SAPS-II |
| Elke 2018 | ICU mortality (no-survival), no details provided | Mortality/general, no details | LOW | HIGH | MODERATE | MODERATE | MODERATE | HIGH | Age, presence of comorbidities and septic shock, lactate, SOFA, APACHE-II, SAPS-II |
| Elke 2018 | Hospital mortality (no-survival), no details provided | Mortality/general, no details | LOW | HIGH | MODERATE | MODERATE | MODERATE | HIGH | Age, presence of comorbidities and septic shock, lactate, SOFA, APACHE-II, SAPS-II |
| Erdogan 2021 | Mortality during the first 28-30 days, no details provided | Mortality/general at 28-30 days | LOW | MODERATE | MODERATE | MODERATE | MODERATE | MODERATE | APACHE II score, levels of SOFA, platelet,lactate and SCUBE-1 |
| Guo 2018 | Mortality during the first 28-30 days, no details provided | Mortality/general at 28-30 days | HIGH | MODERATE | LOW | MODERATE | HIGH | HIGH | arterial lactate and NT-proBNP |
| Hu 2018 | All-cause mortality at 28-days | Mortality/general at 28-30 days | MODERATE | MODERATE | MODERATE | MODERATE | HIGH | HIGH | Not adjusted |
| Jain 2014 | Sepsis-related hospital mortality measured at 28-30 days | Mortality/general at 28-30 days | LOW | LOW | MODERATE | MODERATE | MODERATE | HIGH | Not adjusted |
| Jekarl 2019 | In-hospital mortality, no details | Mortality/general, no details | LOW | MODERATE | MODERATE | LOW | LOW | HIGH | Not adjusted |
| Li 2019 | ICU mortality, no details provided | Mortality/general, no details | LOW | LOW | LOW | MODERATE | LOW | MODERATE | Age, APACHE II, Log-NT-pro-BNP, Lactic acid, HCT, NEU, Prealbumin |
| Liu 2021 | Mortality during the first 28-30 days, no details provided | Mortality/general at 28-30 days | MODERATE | LOW | low | MODERATE | LOW | LOW | age, sex, BMI, SBP, APACHE II, and SOFA scores. |
| Ryoo 2019 | Mortality measured at 28-30 days | Mortality/general at 28-30 days | HIGH | MODERATE | MODERATE | LOW | LOW | HIGH | age, pneumonia, UTI, SOFA, APACHE-II, creatinine, lactate level |
| Song 2019 | Mortality measured at 28-30 days | Mortality/general at 28-30 days | MODERATE | MODERATE | MODERATE | MODERATE | MODERATE | HIGH | Not adjusted |
| Su 2013 | Mortality measured at 28-30 days | Mortality/general at 28-30 days | LOW | MODERATE | MODERATE | MODERATE | LOW | HIGH | Sex, age, temperature, serum sTREM-1, WBC, APACHE-II, SOFA, use of life support technology, etiological factors, pathogens |
| Suberviola 2013 | In-hospital mortality, no details | Mortality/general, no details | LOW | MODERATE | MODERATE | MODERATE | LOW | HIGH | Sex, age, immunosupression status, APACHE-II |
| Sun 2011 | Mortality measured at 28-30 days | Mortality/general at 28-30 days | LOW | MODERATE | MODERATE | MODERATE | MODERATE | MODERATE | sTREM-1, b APACHE 11 score, WBC |
| Viallon 2008 | Mortality measured at 28-30 days | Mortality/general at 28-30 days | MODERATE | MODERATE | MODERATE | MODERATE | MODERATE | HIGH | SAPS-II, lactate |
| Xie00202021 | Sepsis-related mortality measured at 28-30 days | Mortality/general at 28-30 days | LOW | LOW | MODERATE | MODERATE | HIGH | HIGH | neutrophil-to-WBC, lactate |
| Zhao 2013 | 28 day mortality | Mortality/general at 28-30 days | LOW | LOW | LOW | LOW | LOW | LOW | MEDS score and age |
| Zhao J 2020 | Mortality measured at 28-30 days | Mortality/general at 28-30 days | MODERATE | MODERATE | HIGH | LOW | MODERATE | HIGH | Heart rate, Systolic blood pressure, SOFA, leukocyte and serum ammonia |
| Huang 2022 | 28 day mortality | Mortality/general at 28-30 days | MODERATE | MODERATE | LOW | LOW | LOW | LOW | age, sex, comorbidities, site of infection,lymphocytes, platelets, APACHE II and SOFA score, |
| Kim 2022 | 28-day mortality | Mortality/general at 28-30 days | MODERATE | MODERATE | LOW | MODERATE | LOW | MODERATE |  |
| Koozi 2023 | 30-day mortality | Mortality/general at 28-30 days | MODERATE | LOW | MODERATE | MODERATE | LOW | MODERATE | Age, GCs, Temperatura, CRP, Inmunosupresion, Biliirubina |
| Tokur 2022 | 28-day mortality | Mortality/general at 28-30 days | LOW | LOW | LOW | LOW | HIGH | HIGH | Red Cell Distribution Width |

**IL-6 assessments**

| **Study** | **Outcome** | **Outcome category** | **D1-Study participation: risk of bias rating (high/low/moderate)** | **D2-Study attrition: risk of bias rating (high/low/moderate)** | **D3-Biomarker measurement: risk of bias rating (high/low/moderate)** | **D4-Outcome measurement: risk of bias rating (high/low/moderate)** | **D5-Adjustment for other prognostic factors: risk of bias rating (high/low/moderate)** | **D6-Statistical analysis and reporting: risk of bias rating (high/low/moderate)** | **Model adjusted by** |
| --- | --- | --- | --- | --- | --- | --- | --- | --- | --- |
| Amancio 2013 | Hospital mortality (no-survival), no details provided | Mortality/general, no details | MODERATE | HIGH | MODERATE | MODERATE | LOW | HIGH | Age, Severe comorbidity and SOFA score, Immunosupression (HIV diagnosis) |
| Andaluz-Ojeda 2012 | ICU mortality during the first 28-30 days | Mortality/general at 28-30 days | LOW | MODERATE | MODERATE | MODERATE | MODERATE | HIGH | APACHE II, other biomarkers |
| Eidt 2016 | Mortality, no details provided | Mortality/general, no details | MODERATE | MODERATE | MODERATE | MODERATE | HIGH | HIGH | age, sex, lactate, other biomarkers |
| Jiang 2019 | Mortality during the first 28-30 days, no details provided | Mortality/general at 28-30 days | LOW | MODERATE | LOW | MODERATE | MODERATE | HIGH | Plasma levels of EPO, hepcidin, ferritin, sTfR/log ferritin as well as the RDW and SOFA score |
| Liu 2021 | Mortality during the first 28-30 days, no details provided | Mortality/general at 28-30 days | MODERATE | LOW | low | MODERATE | LOW | LOW | age, sex, BMI, SBP, APACHE II, and SOFA scores. |
| Oberholzer 2005 | Mortality measured at 28-30 days | Mortality/general at 28-30 days | HIGH | MODERATE | LOW | LOW | LOW | HIGH | APACHE-II. Age, treatments, baseline MOD |
| Phua 2008 | Mortality measured at 28-30 days | Mortality/general at 28-30 days | LOW | LOW | LOW | MODERATE | MODERATE | HIGH | APACHE II, SOFA scores, IL-1B, IL 10 and lactate levels |
| Siddiqui 2019 | All-cause mortality during the first 28-30 days | Mortality/general at 28-30 days | MODERATE | MODERATE | HIGH | MODERATE | LOW | MODERATE | baseline age, gender, surgical method and Qsofa. |
| Song 2019 | Mortality measured at 28-30 days | Mortality/general at 28-30 days | MODERATE | MODERATE | MODERATE | MODERATE | MODERATE | HIGH | APACHE-II, SOFA, pentraxin, lactate, septi shock |
| Viallon 2008 | Mortality measured at 28-30 days | Mortality/general at 28-30 days | MODERATE | MODERATE | MODERATE | MODERATE | MODERATE | HIGH | SAPS-II, lactate |
| Wu C 2021 | ICU mortality during the first 28-30 days | Mortality/general at 28-30 days | HIGH | LOW | LOW | MODERATE | MODERATE | MODERATE | SOFA and IL37 |
| Wu H 2009 | Mortality measured at 28-30 days | Mortality/general at 28-30 days | LOW | MODERATE | MODERATE | MODERATE | MODERATE | HIGH | APACHE II, septic shock, gas-trointestinal bleeding, and plasma IL-6, IL-10 andTGF-β1 |
| Xie 2021 | Sepsis-related mortality measured at 28-30 days | Mortality/general at 28-30 days | LOW | LOW | MODERATE | MODERATE | HIGH | HIGH | neutrophil-to-WBC, lactate |
| Zhang 2019 | 28 day mortality | Mortality/general at 28-30 days | HIGH | MODERATE | MODERATE | MODERATE | MODERATE | HIGH | SOFA, PCT, NT-proBNP,Prothombin time, thrombin time |
| Zhao 201 | 28 day mortality | Mortality/general at 28-30 days | LOW | LOW | LOW | LOW | LOW | LOW | MEDS score and age |
| Belli 2022 | 30 day mortality | Mortality/general at 28-30 days | LOW | MODERATE | MODERATE | MODERATE | HIGH | HIGH | Univariable |
| Karamouzos 2021 | 28-day mortality | Mortality/general at 28-30 days | MODERATE | LOW | LOW | MODERATE | MODERATE | HIGH | Sexo, microorganismo, suceptibilidad, tipode infeccion, diabetes, Citokinas |
| Karampela 2022 | 28-day mortality | Mortality/general at 28-30 days | HIGH | MODERATE | LOW | MODERATE | MODERATE | HIGH | Cheperin APACHE PCT |
| Lai 2022 | 28-day mortality | Mortality/general at 28-30 days | LOW | LOW | LOW | MODERATE | MODERATE | HIGH | HE4, SOFA, PCT |
| Xie 2023 | 28-day mortality | Mortality/general at 28-30 days | LOW | MODERATE | LOW | MODERATE | HIGH | HIGH | IL-6 PCT dia 1 y dia 3 Lactato |
| Yu 2023 | 28-day mortality | Mortality/general at 28-30 days | LOW | LOW | LOW | LOW | LOW | LOW | Sofa IL-6 LACT BUN Age |
| Zang LT 2022 | ICU mortality | Mortality/general, no details | LOW | MODERATE | MODERATE | MODERATE | MODERATE | HIGH | NSE, APACHE |

**sCD14 assessments**

| **Study** | **Outcome** | **Outcome category** | **D1-Study participation: risk of bias rating (high/low/moderate)** | **D2-Study attrition: risk of bias rating (high/low/moderate)** | **D3-Biomarker measurement: risk of bias rating (high/low/moderate)** | **D4-Outcome measurement: risk of bias rating (high/low/moderate)** | **D5-Adjustment for other prognostic factors: risk of bias rating (high/low/moderate)** | **D6-Statistical analysis and reporting: risk of bias rating (high/low/moderate)** | **Model adjusted by** |
| --- | --- | --- | --- | --- | --- | --- | --- | --- | --- |
| Aalto 2007 | Hospital mortality during the first 28-30 days | Mortality/general at 28-30 days | MODERATE | HIGH | HIGH | MODERATE | HIGH | HIGH | Age and gender |
| Masson 2014 | Mortality during the first 28-30 days | Mortality/general at 28-30 days | LOW | MODERATE | LOW | MODERATE | LOW | MODERATE | SAPS II score, SOFA score, Serum lactate concentratio, mean arterial pressure, central venous oxygen saturation and randomized treatment (matched by age and sex) |
| Masson 2014 | ICU mortality, no details provided | Mortality/general, no details | LOW | MODERATE | LOW | MODERATE | LOW | MODERATE | SAPS II score, SOFA score, Serum lactate concentratio, mean arterial pressure, central venous oxygen saturation and randomized treatment (matched by age and sex) |
| Khashab 2022 | 28-day mortality | Mortality/general at 28-30 days | LOW | MODERATE | LOW | MODERATE | MODERATE | HIGH | SOFA, NEWS2 PCT |
| Lee J 2021 | in-hospital mortality, no details | Mortality/general, no details | HIGH | MODERATE | MODERATE | MODERATE | HIGH | HIGH |  |
| Lee S 2022 | 30 day mortality | Mortality/general at 28-30 days | LOW | MODERATE | MODERATE | MODERATE | MODERATE | MODERATE | SOFA, Apache lactato, shock septico, WBC ; Bilirrubin Platetel |
| Shymoyama 2021 | 28-day mortality | Mortality/general at 28-30 days | LOW | MODERATE | MODERATE | MODERATE | LOW | HIGH |  |

**S6 Table.** Baseline PCT values and mortality: measure effects

|  | **Number of studies (deaths/Total)** | **Adjustment by** | **Measure of effect (95% CI)** |
| --- | --- | --- | --- |
| **Mortality at 28-30 days** | | | |
| **Categorical** |  | | |
| Threshold ≥ 4270 pg/ml | 1 study (NR/360) | Age, severity score or other covariates | OR = 0.89 (0.82 to 0.97) |
| Threshold ≥ 500 pg/ml | 1 study (173/821) | Age, severity score or other covariates | OR = 10.9 (5.38 to 22.1) |
| Threshold ≥ 3870 pg/ml | 1 study (134/501) | Age, severity score or other covariates | OR = 1.09 (1.03 to 1.16) |
| Quartiles | 1 study (46/220) | Age, severity score or other covariates | HR = 1.42 (1.08 to 1.86) |
| Threshold ≥ 7000 pg/ml | 1 study (131/228) | Age, severity score or other covariates | RR = 1.55 (1.07 to 2.25) |
| **Continuous** |  | | |
| Individual HR – adjusted | 1 study (57/205) | Both age and a severity score | HR = 1.04 (1.01 to 1.07) |
| Pooled HR – partially or no adjusted | 9 studies (>665/>2344) | Age, severity score or other covariates | HR = 1.16 (0.95 to 1.40)^a^ |
| HR Narrative | 2 studies (66/154) | Age, severity score or other covariates | No significant (p-values) |
| Pooled OR – adjusted | 3 studies (409/1129) | Both age and a severity score | OR = 0.99 (0.99 to 1.00) ^b^ |
| Pooled OR – partially or no adjusted | 11 studies (927/3565) | Age, severity score or other covariates | OR = 1.03 (0.85 to 1.24) ^c^ |
| OR Narrative | 2 studies (43/131) | Age, severity score or other covariates | No significant (p-values) |
| **Log-transformed** |  | | |
| log (ng/mL) | 1 study (101/326) | Age, severity score or other covariates | HR = 1.1 (0.9 to 1.4) |
| log (µg/mL) | 1 study (50/100) | Both age and a severity score | HR = 0.97 (0.84 to 1.12) |
| **Mortality, no details** | | | |
| **Categorical** |  | | |
| Threshold ≥ 830 pg/ml | 1 study (41/137) | Both age and a severity score | OR = 2.70 (0.48 to 15.30) |
| Threshold ≥ 2000 pg/mL | 1 study (NR/148) | Age, severity score or other covariates | OR = 3.67 (1.54 to 8.70) |
| **Continuous** |  | | |
| Individual HR | 1 study (289/1076) | Age, severity score or other covariates | HR = 1.60 (1.30 to 2.10) ^d^ |
| Pooled OR – partially or no adjusted | 2 study (155/510) | Age, severity score or other covariates | OR = 0.98 (0.97 to 1.01)^e^ |
| **Log-transformed** |  | | |
| log (µg/mL) | 1 study (50/100) | Both age and a severity score | HR = 0.89 (0.75 to 1.05) |
| log (ng/mL) | 1 study (26/185) | Age, severity score or other covariates | OR = 2.01 (1.24 to 3.23) |

**Notes:** a) I-squared= 99.84%, P=0.00; predictive interval 95%= 0.39, 3.66; b) I-squared= 4.88%, P=0.730; predictive interval 95%= 0.97, 1.03; c) I-squared= 99.96%, P<0.01; predictive interval 95%= 0.48, 2.01; d) Elke 2018 provided 2 assessments (ICU mortality and hospital mortality), the bigger measure effect is showed in the table; e) I-squared= 0.00%, P=0.974; predictive interval 95%= No estimable.

**S1 Figure.** Procalcitonin and prediction of mortality at 28-30 days in critically-ill septic patients


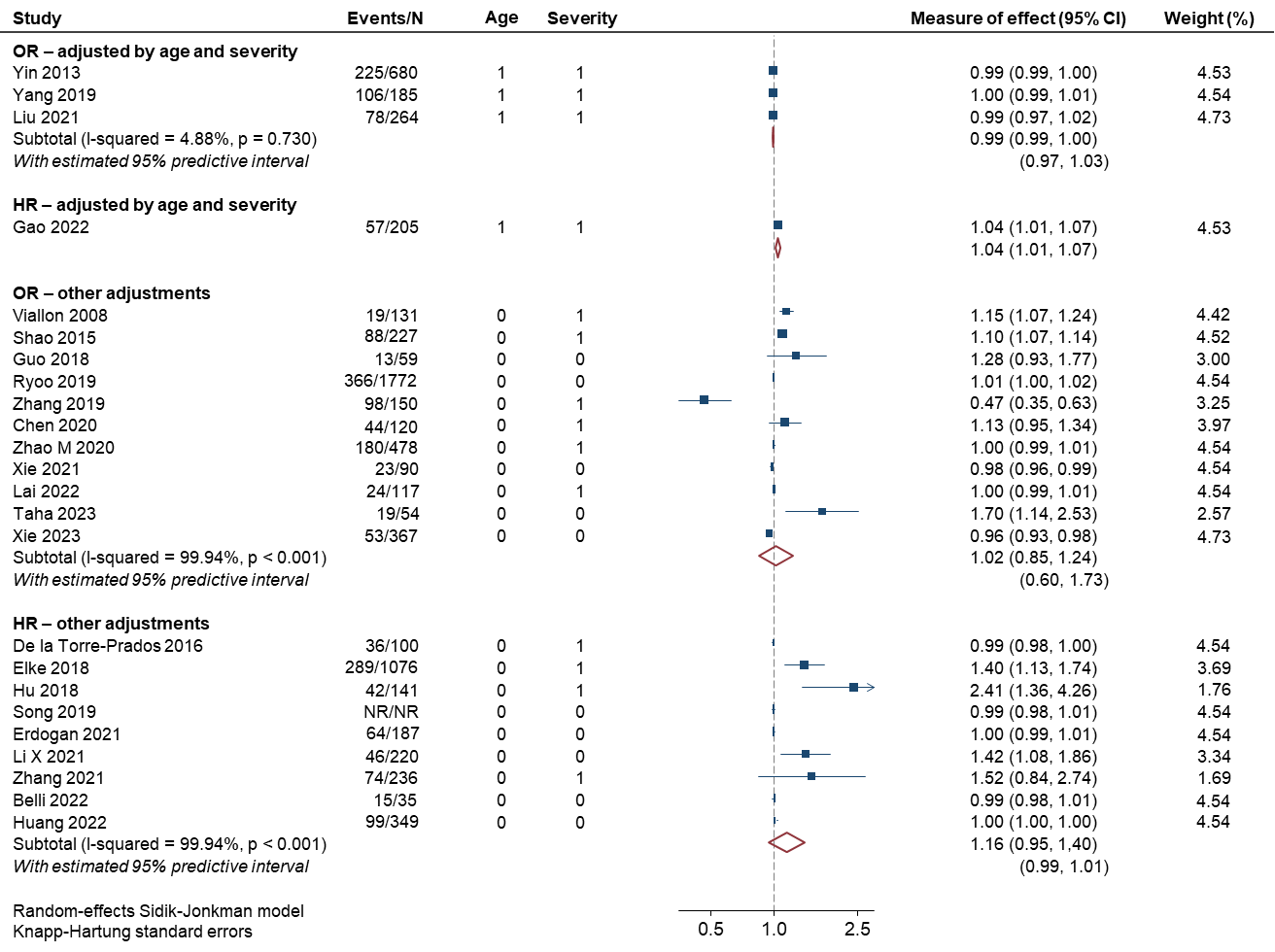


**S7 Table.** Baseline CRP values and mortality: measure effects

|  | **Number of studies (deaths/Total)** | | | **Adjustment by** | **Measure of effect (95% CI)** |
| --- | --- | --- | --- | --- | --- |
| **Mortality at 28-30 days** | | | | | |
| **Categorical** |  | | | | |
| Threshold ≥ 27.5 mg/L | 1 study (134/501) | | | Both age and a severity score | OR = 1.00 (0.99 to 1.01) |
| **Continuous** |  | | | | |
| Pooled HR – adjusted | | 2 (188/631) | Both age and a severity score | | HR = 1.01 (0.92 to 1.11)^a^ |
| Pooled HR – partially or no adjusted | 6 studies (>505/>1740) | | | Age, severity score or other covariates | HR = 1.03 (0.96 to 1.10)^b^ |
| HR Narrative | 2 studies (66/154) | | | Age, severity score or other covariates | No significant (p-values) |
| Pooled OR – adjusted | 2 study (103/303) | | | Both age and a severity score | OR = 1.01 (0.87 to 1.17)^c^ |
| Pooled OR – partially or no adjusted | 5 studies (513/2287) | | | Age, severity score or other covariates | OR = 1.01 (0.99 to 1.03)^d^ |
| OR Narrative | 1 study (19/131) | | | Age, severity score or other covariates | No significant (p-values) |
| **Log-transformed** |  | | | | |
| log (mg/dL) | 1 study (101/326) | | | Age, severity score or other covariates | HR = 1.30 (0.80 to 1.90) |
| **Mortality, no details** | | | | | |
| **Categorical** |  | | | | |
| Threshold ≥ 3.55 mg/dL | 1 study (41/137) | | | Both age and a severity score | No significant (p-values) |
| **Continuous** |  | | | | |
| Individual HR | 1 study (289/1076) | | | Age, severity score or other covariates | HR = 1.20 (1.00 to 1.40) ^e^ |
| OR Narrative | 2 studies (143/607) | | | Age, severity score or other covariates | No significant (p-values) |
| **Log-transformed** |  | | | | |
| log (mg/L) | 1 study (62/245) | | | Both age and a severity score | OR = 1.81 (1.20 to 2.72) |

**Notes:** a) I-squared = 57.74%, P = 0.116; predictive interval 95% = Not estimable; b) I-squared = 98.77%, P = 0.238; predictive interval 95%= 0.99 to 1.02; c) I-squared = 61.76%, P = 0.096; predictive interval 95% = Not estimable; d) I-squared = 95.41%, P = 0.004; predictive interval 95% = 0.98 to 1.03; e) Elke 2018 provided 2 assessments (ICU mortality and hospital mortality), the bigger measure effect is showed in the table

**S2 Figure.** C-reactive protein and prediction of mortality at 28-30 days in critically-ill septic patients


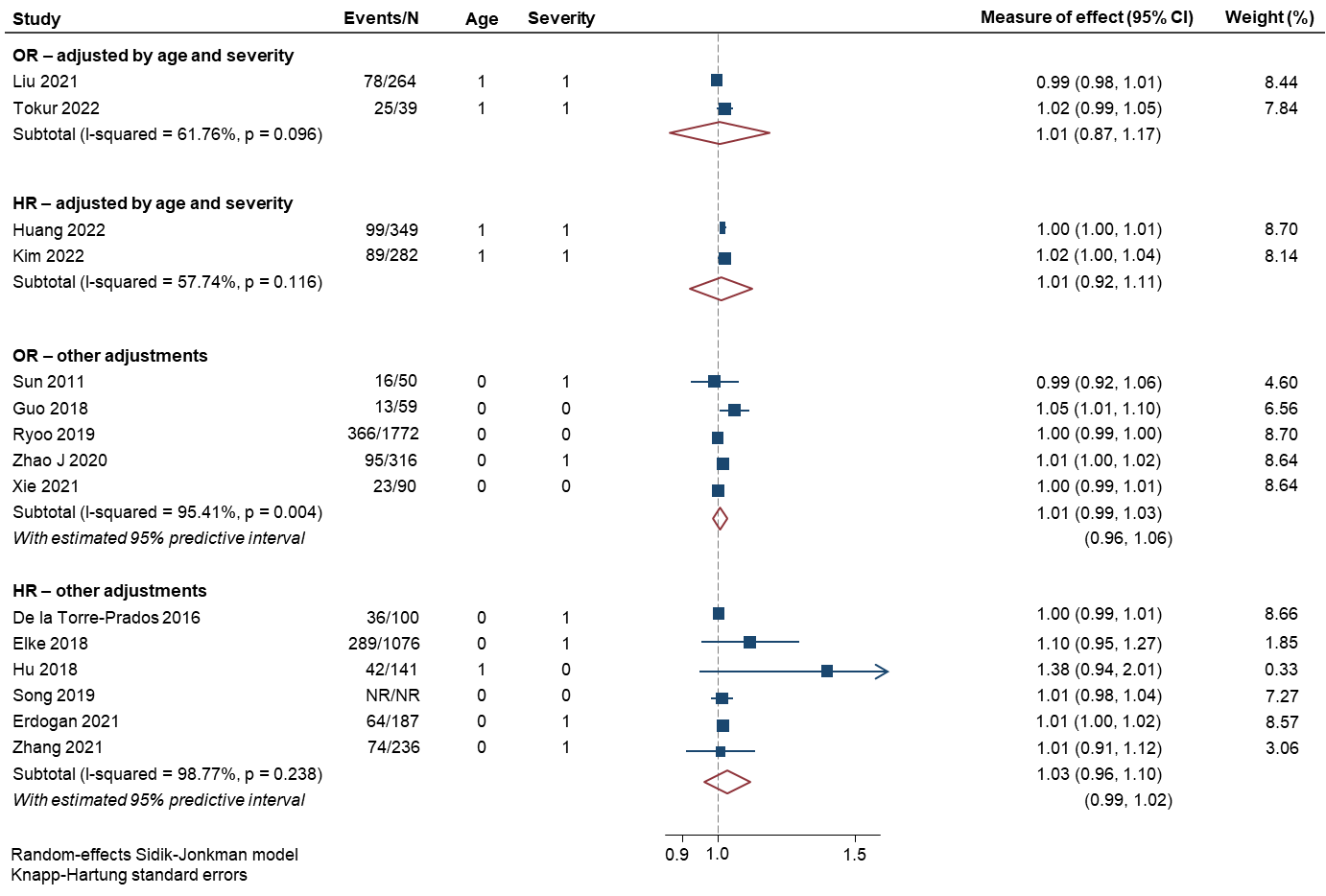


**S8 Table.** Baseline IL-6 values and mortality: measure effects

|  | **Number of studies (deaths/Total)** | **Adjustment by** | **Measure of effect (95% CI)** |
| --- | --- | --- | --- |
| **Mortality at 28-30 days** | | | |
| **Categorical** |  | | |
| Threshold ≥ 201.68 pg/mL | 1 study (51/114) | Age, severity score or other covariates | OR = 1.66 (0.67 to 4.10) |
| Threshold ≥ 22.8 pg/mL | 1 study (134/501) | Both age and a severity score | OR = 1.00 (1.00 to 1.00) |
| **Log-transformed** |  | | |
| log (Threshold ≥ 867.2 pg/mL) | 1 study (12/29) | Age, severity score or other covariates | HR = 2.00 (1.22 to 3.27) |
| **Continuous** |  | | |
| Individual HR | 1 study (NR/97) | Age, severity score or other covariates | HR = 1.00 (1.00 to 1.00) |
| Individual OR– adjusted | 1 study (78/264) | Both age and a severity score | OR = 1.02 (1.01 to 1.03) |
| Pooled OR– partially or no adjusted | 7 studies (>224/>963) | Age, severity score or other covariates | OR = 1.00 (0.99 to 1.01)^a^ |
| OR Narrative | 3 studies (70/251) | Age, severity score or other covariates | No significant (p-values) |
| **Log-transformed** |  | | |
| log (pg/mL+log) | 1 study (NR/198) | Both age and a severity score | HR = 1.46 (1.11 to 1.92) |
| log (pg/mL+log) | 1 study (39/124) | Both age and a severity score | No significant (p-values) |
| **Mortality, no details** | | | |
| **Log-transformed** |  | | |
| log (pg/mL) | 1 study (33/60) | Both age and a severity score | OR = 1.00 (1.00 to 1.01) |
| log (pg/mL) | 1 study (18/75) | Age, severity score or other covariates | OR = 1.00 (1.00 to 1.00) |

**Notes:** a) I-squared= 99.52%, P=0.270; predictive interval 95%= 0.98 to 1.03.

**S3 Figure.** Interleukin-6 and prediction of mortality at 28-30 days in critically-ill septic patients


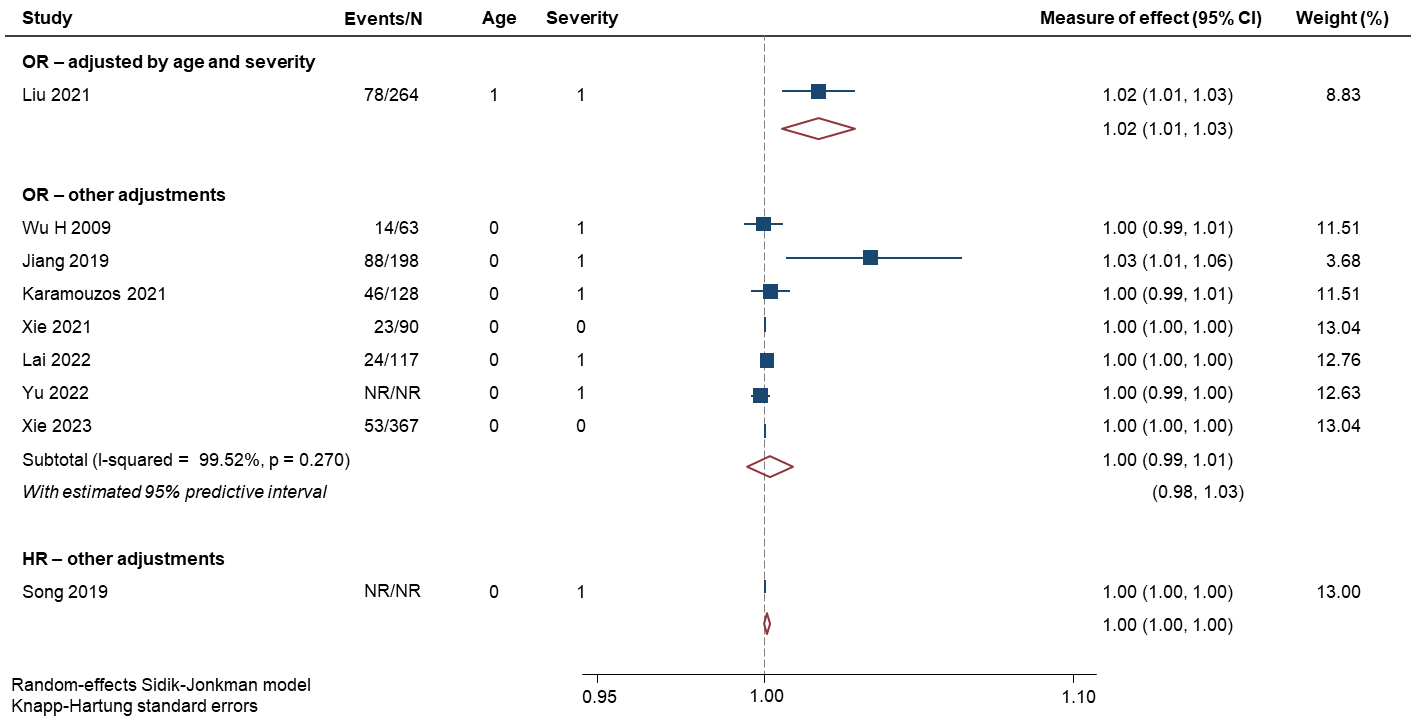


**S9 Table.** Baseline sCD14 values and mortality: measure effects

|  | **Number of studies (deaths/Total)** | **Adjustment by** | **Measure of effect (95% CI)** |
| --- | --- | --- | --- |
| **Mortality at 28-30 days** | | | |
| **Categorical** |  | | |
| Threshold ≥ highest tertile, no details provided | 1 study (10/142) | Age, severity score or other covariates | HR= 1.22 (0.20 to 5.47) |
| **Continuous** |  |  |  |
| Pooled HR | 2 (>74/>278) | Age, severity score or other covariates | HR = 1.00 (1.00 to 1.01)^a^ |
| Individual unclear model | 1 (41/178) | Age, severity score or other covariates | Unclear = 1.28 (1.09 to 1.49) |
| **Log-transformed** |  | | |
| log (pg/mL) | 1 study (50/100) | Both age and a severity score | HR = 1.55 (1.12 to 2.13)^b^ |
| **Mortality, no details** | | | |
| **Log-transformed** |  | | |
| log (pg/mL) | 1 study (50/100) | Both age and a severity score | HR = 1.51 (1.05 to 2.17)^b^ |

**Notes:** a) I-squared = 0.43%, P = 0.757; predictive interval 95% = Not estimable; b) Masson 2017 provided two analysis, one for mortality at 28-30 days and another for mortality-no details.

**S4 Figure.** sCD14 and prediction of mortality at 28-30 days in critically-ill septic patients


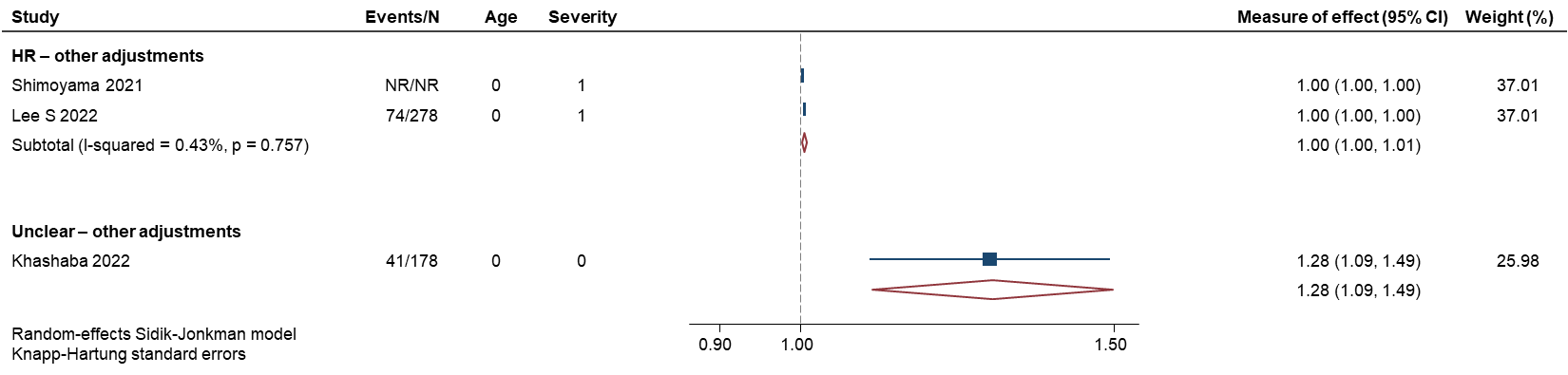


**S4 Figure**. Summary of mortality at 28-30 days and baseline biomarkers measures


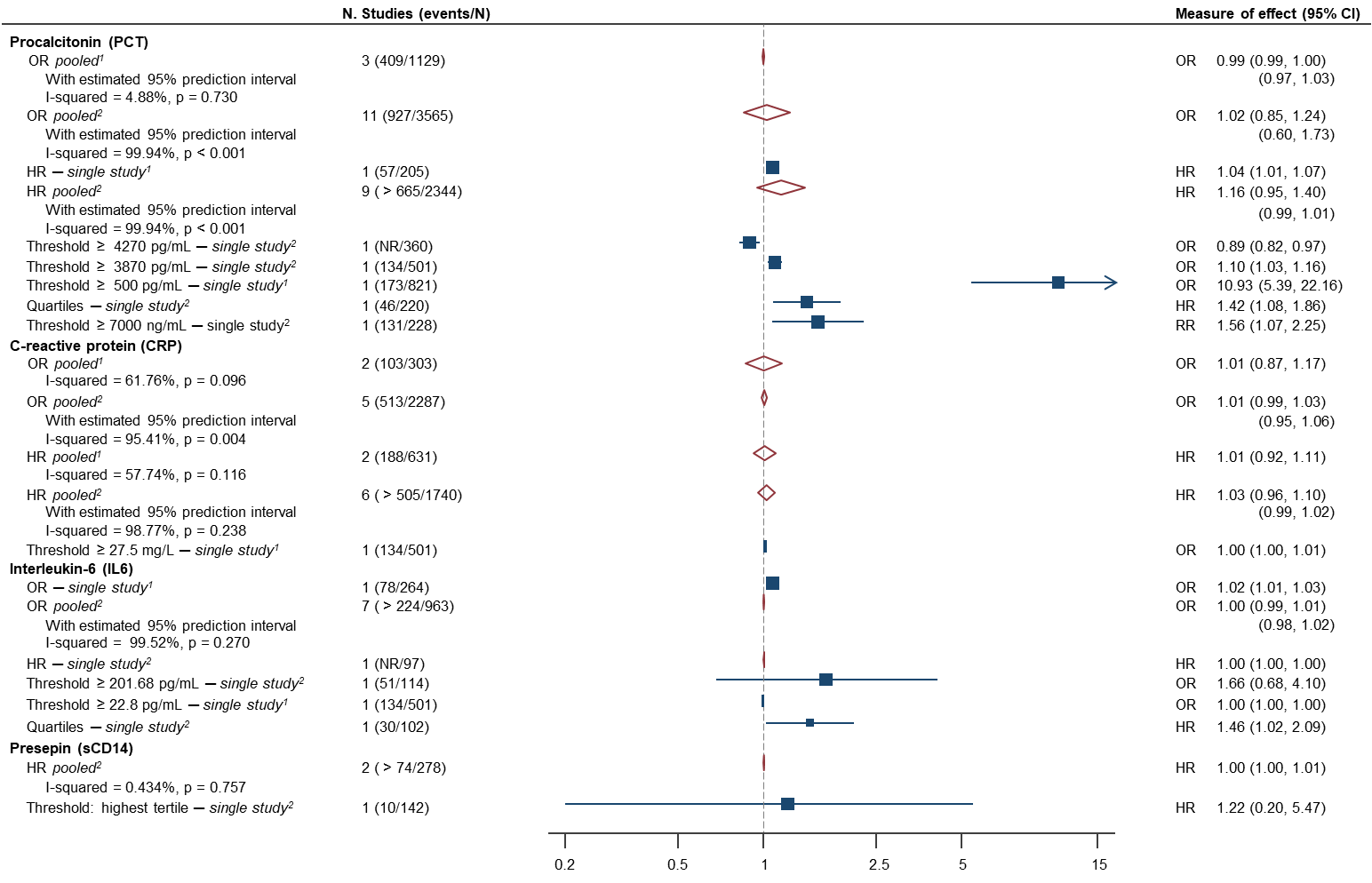


**Notes**: 1) Measure of effect adjusted by both sex and a severity score plus other covariates; 2) measure of effect adjusted by sex, a severity score, or by other covariates; 3) two additional studies evaluating 131 septic patients and 43 deaths found no statistical significantly OR values (narrative report); 4) two additional studies evaluating 154 septic patients and 66 deaths found no statistical significantly HR values (narrative report); 5) one additional study evaluating 131 septic patients and 19 deaths found no statistical significantly OR values (narrative report); 6) two additional studies evaluating 154 septic patients and 66 deaths found no statistical significantly HR values (narrative report).

**S5 Figure**. Summary of mortality, no details provided and baseline biomarkers measures


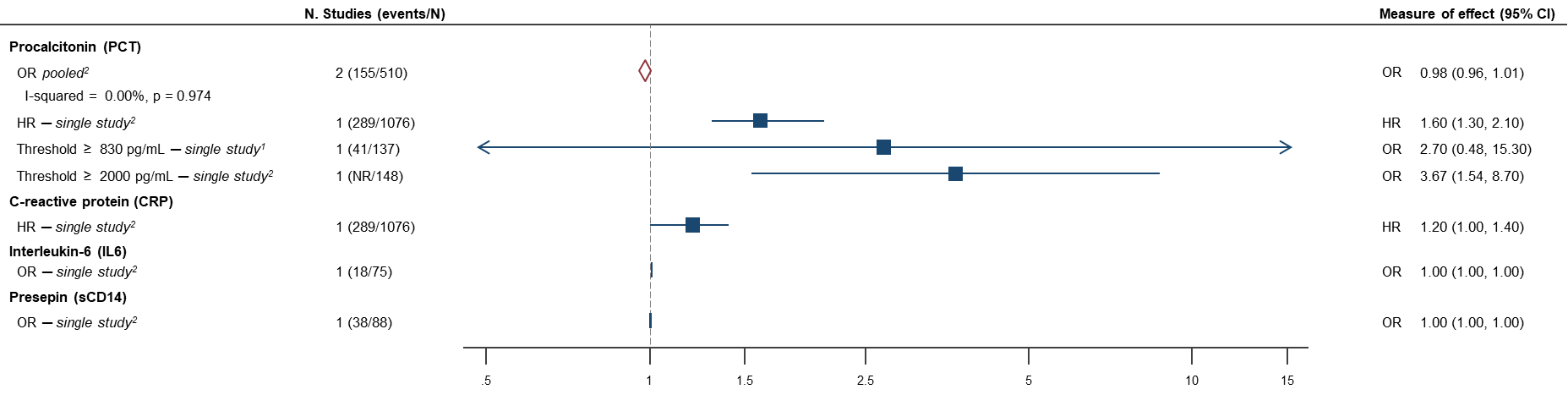


**Notes**: 1) Measure of effect adjusted by both sex and a severity score plus other covariates; 2) measure of effect adjusted by sex, a severity score, or by other covariates. More info in Supplements

**EXCLUDED REFERENCES**

1. Azevedo JRAd, Torres OJM, Czeczko NG, Tuon FF, Nassif PAN, Souza GDd. Procalcitonin as a prognostic biomarker of severe sepsis and septic shock. *Revista do Colegio Brasileiro de Cirurgioes* 2012; **39**(6): 456-61.

2. Calandra T, Gerain J, Heumann D, Baumgartner JD, Glauser MP. High circulating levels of interleukin-6 in patients with septic shock: evolution during sepsis, prognostic value, and interplay with other cytokines. The Swiss-Dutch J5 Immunoglobulin Study Group. *The American journal of medicine* 1991; **91**(1): 23-9.

3. Gao C, Chen H. Correlation of serum miR-127 level with severity and prognosis of sepsis. *American Journal of Translational Research* 2022; **14**(11): 7994-8001.

4. Gao Q, Cheng Y, Li Z, et al. Association between nutritional risk screening score and prognosis of patients with sepsis. *Infection and Drug Resistance* 2021; **14**: 3817-25.

5. Di S, Wang Y, Sun L, Zhao H, Guo L. The value of TLR-4, CRP, PCT and WBC levels in assessing the diagnosis and prognosis of sepsis patients. *International Journal of Clinical and Experimental Medicine* 2020; **13**(12): 9419-28.

6. El Said AM, Fayed AM, El-Reweny EM. Comparative study between complete blood picture indices and presepsin as early prognostic markers in septic shock patients. *Egyptian Journal of Anaesthesia* 2020; **36**(1): 118-25.

7. Hashim RM, Abdelaziz MM, Hassan RM. Higher accuracy of concurrent use of corrected QT interval and procalcitonin serum level to predict sepsis related mortality in ICU patients. *Egyptian Journal of Anaesthesia* 2020; **36**(1): 38-43.

8. Hillas G, Vassilakopoulos T, Plantza P, Rasidakis A, Bakakos P. C-reactive protein and procalcitonin as predictors of survival and septic shock in ventilator-associated pneumonia. *The European respiratory journal* 2010; **35**(4): 805-11.

9. Honorato MO, Filho JTS, Honorato Junior LFB, Watanabe N, Goulart GM, Do Prado RR. Atrial Fibrillation and Sepsis in Elderly Patients and Their Associaton with In-Hospital Mortality. *Arquivos Brasileiros de Cardiologia* 2023; **120**(3).

10. Kandaswamy P, Hemlata, Singh GP, Ahmad MK. Comparative evaluation of procalcitonin and interleukin-6 as diagnostic and prognostic biomarkers for sepsis. *Journal of Clinical and Diagnostic Research* 2018; **12**(10): UC17-UC21.

11. Kang FX, Wang RL, Yu KL, Wei Q. The study on pro-adrenomedullin as a new biomarker in sepsis prognosis and risk stratification. *Chinese Critical Care Medicine* 2008; **20**(8): 452-5.

12. Karampela I, Christodoulatos GS, Kandri E, et al. Circulating eNampt and resistin as a proinflammatory duet predicting independently mortality in critically ill patients with sepsis: A prospective observational study. *Cytokine* 2019; **119**: 62-70.

13. Kellum JA, Kong L, Fink MP, et al. Understanding the inflammatory cytokine response in pneumonia and sepsis: Results of the genetic and inflammatory markers of sepsis (GenIMS) study. *Archives of Internal Medicine* 2007; **167**(15): 1655-63.

14. Kim CH, Park JT, Kim EJ, et al. An increase in red blood cell distribution width from baseline predicts mortality in patients with severe sepsis or septic shock. *Critical Care* 2013; **17**(6).

15. Li Y, Wang J, Wei B, Zhang X, Hu L, Ye X. Value of Neutrophil:Lymphocyte Ratio Combined with Sequential Organ Failure Assessment Score in Assessing the Prognosis of Sepsis Patients. *International Journal of General Medicine* 2022; **15**: 1901-8.

16. Li Z, Luo Z, Shi X, Pang B, Ma Y, Jin J. The Levels of Oxidized Phospholipids in High-Density Lipoprotein During the Course of Sepsis and Their Prognostic Value. *Frontiers in Immunology* 2022; **13**.

17. Nanda SK, Dinakaran A, Sandhya Bhat K, Ravichandran, Kanungo R. Diagnostic and prognostic role of Procalcitonin in sepsis in a tertiary care hospital. *Biomedical Research (India)* 2016; **27**(1): 79-83.

18. Pieralli F, Vannucchi V, Mancini A, et al. Procalcitonin Kinetics in the First 72 Hours Predicts 30-Day Mortality in Severely Ill Septic Patients Admitted to an Intermediate Care Unit. *Journal of clinical medicine research* 2015; **7**(9): 706-13.

19. Schroder J, Staubach KH, Zabel P, Stuber F, Kremer B. Procalcitonin as a marker of severity in septic shock. *Langenbeck's archives of surgery* 1999; **384**(1): 33-8.

20. Schuetz P, Christ-Crain M, Morgenthaler NG, Struck J, Bergmann A, Muller B. Circulating precursor levels of endothelin-1 and adrenomedullin, two endothelium-derived, counteracting substances, in sepsis. *Endothelium : journal of endothelial cell research* 2007; **14**(6): 345-51.

21. Sharma R, Vijayakumar M. Procalcitonin for improved assessment and an answer to sepsis dilemma in critically ill - a myth, a hype, or a reality ? *Nitte university journal of health science* 2014; **4**(1): 57‐65.

22. Stalder G, Que YA, Calzavarini S, et al. Study of Early Elevated Gas6 Plasma Level as a Predictor of Mortality in a Prospective Cohort of Patients with Sepsis. *PloS one* 2016; **11**(10): e0163542.

23. Su L-x, Meng K, Zhang X, et al. Diagnosing ventilator-associated pneumonia in critically ill patients with sepsis. *American journal of critical care : an official publication, American Association of Critical-Care Nurses* 2012; **21**(6): e110-9.

24. Sunden-Cullberg J, Norrby-Teglund A, Rouhiainen A, et al. Persistent elevation of high mobility group box-1 protein (HMGB1) in patients with severe sepsis and septic shock. *Critical care medicine* 2005; **33**(3): 564-73.

25. Tan Y, Zhou K, Tang X, et al. Bacteremic and non-bacteremic pneumonia caused by Acinetobacter baumannii in ICUs of South China: A Clinical and Microbiological Study. *Scientific reports* 2017; **7**(1): 15279.

26. Thao PTN, Tra TT, Son NT, Wada K. Reduction in the IL-6 level at 24 h after admission to the intensive care unit is a survival predictor for Vietnamese patients with sepsis and septic shock: a prospective study. *BMC emergency medicine* 2018; **18**(1): 39.

27. Yao L, Liu Z, Zhu J, Li B, Chai C, Tian Y. Higher serum level of myoglobin could predict more severity and poor outcome for patients with sepsis. *The American journal of emergency medicine* 2016; **34**(6): 948-52.

28. Zhang L, Qiu C, Yang L, et al. GPR18 expression on PMNs as biomarker for outcome in patient with sepsis. *Life sciences* 2019; **217**: 49-56.

29. Baoquan W, Nan L, Haiyan F, Zhansheng H. Analysis of prognostic factors in patients with sepsis and septic shock in intensive care unit. *Chinese Journal of Infection and Chemotherapy* 2019; **19**(5): 499-504.

30. Malek F, Gohari A, Mirmohammadkhani M, Ardiani F. Relationship between the serum level of C-reactive protein and severity and outcomes of community-acquired pneumonia. *Archives of Clinical Infectious Diseases* 2019; **14**(2).

31. Naderpour Z, Momeni M, Vahidi E, Safavi J, Saeedi M. Procalcitonin and D-dimer for Predicting 28-Day-Mortality Rate and Sepsis Severity based on SOFA Score; A Cross-sectional Study. *Bulletin of emergency and trauma* 2019; **7**(4): 361-5.

32. Peng JC, Xu QY, Ding J, et al. Usefulness of procalcitonin clearance to predict mortality in abdominal sepsis. *European Journal of Inflammation* 2020; **18**.

33. Tschaikowsky K, Hedwig-Geissing M, Braun GG, Radespiel-Troeger M. Predictive value of procalcitonin, interleukin-6, and C-reactive protein for survival in postoperative patients with severe sepsis. *Journal of critical care* 2011; **26**(1): 54-64.

34. Behnes M, Bertsch T, Lepiorz D, et al. Diagnostic and prognostic utility of soluble CD 14 subtype (presepsin) for severe sepsis and septic shock during the first week of intensive care treatment. *Critical care (London, England)* 2014; **18**(5): 507.

35. Frencken JF, van Vught LA, Peelen LM, et al. An Unbalanced Inflammatory Cytokine Response Is Not Associated With Mortality Following Sepsis: A Prospective Cohort Study. *Critical care medicine* 2017; **45**(5): e493-e9.

36. Goldberg I, Shalmon D, Shteinvil R, et al. The superiority of 72 h leukocyte descent over CRP for mortality prediction in patients with sepsis. *Clinica chimica acta; international journal of clinical chemistry* 2021; **514**: 34-9.

37. Gradel KO, Jensen TG, Kolmos HJ, Pedersen C, Vinholt PJ, Lassen AT. Does C-reactive protein independently predict mortality in adult community-acquired bacteremia patients with known sepsis severity? *APMIS : acta pathologica, microbiologica, et immunologica Scandinavica* 2013; **121**(9): 835-42.

38. Kurisu K, Yoshiuchi K, Ogino K, Okada Y, Oda T. Peak C-reactive protein levels do not predict 30-day mortality for bacteremia: A retrospective cohort study. *Journal of infection and chemotherapy : official journal of the Japan Society of Chemotherapy* 2020; **26**(1): 23-7.

39. Matera G, Quirino A, Peronace C, et al. Soluble CD14 Subtype-A New Biomarker in Predicting the Outcome of Critically Ill Septic Patients. *The American journal of the medical sciences* 2017; **353**(6): 543-51.

40. Ogasawara S, Saito N, Hirano R, Minakawa S, Kimura M, Kayaba H. Clinical relevance of procalcitonin values in bacteremia. *Journal of infection and chemotherapy : official journal of the Japan Society of Chemotherapy* 2020; **26**(10): 1048-53.

41. Pallas Beneyto LA, Rodriguez Luis O, Saiz Sanchez C, Coltell O, Bautista Rentero D, Miguel Bayarri V. [Prognostic value of interleukin 6 for death of patients with sepsis]. *Medicina critica* 2016; **147**(7): 281-6.

42. Pauly D, Hamed S, Behnes M, et al. Endothelial cell-specific molecule-1/endocan: Diagnostic and prognostic value in patients suffering from severe sepsis and septic shock. *Journal of critical care* 2016; **31**(1): 68-75.

43. Peng J, Zhang R, Zhao Y, et al. Prognostic value of preoperative prognostic nutritional index and its associations with systemic inflammatory response markers in patients with stage III colon cancer. *Chinese journal of cancer* 2017; **36**(1): 96.

44. Shen YF, Chen LY, He F. A Study on clinical outcomes and death risk factors in adult patients with klebsiella pneumoniae septicemia. *Acta Medica Mediterranea* 2020; **36**(5): 3167-71.

45. Suarez De La Rica A, Maseda E, Anillo V, Hernandez Gancedo C, Lopez-Tofiño A, Gilsanz F. Procalcitonin, c-reactive protein and lactate as prognostic markers of mortality in patients with complicated intraabdominal infection admitted to the ICU. *Intensive Care Medicine* 2013; **39**: S341.

46. Tanriverdi H, Tor MM, Kart L, Altin R, Atalay F, SumbSumbuloglu V. Prognostic value of serum procalcitonin and C-reactive protein levels in critically ill patients who developed ventilator-associated pneumonia. *Annals of thoracic medicine* 2015; **10**(2): 137-42.

47. Taskin G, Ozturk K, Turker T, et al. Assessment of mortality risk factors for criticaly ill patients with acinetobacter baumannii bacteremia with a new perspective. *Acta Medica Mediterranea* 2016; **32**(6): 1892-7.

48. Wunder C, Eichelbronner O, Roewer N. Are IL-6, IL-10 and PCT plasma concentrations reliable for outcome prediction in severe sepsis? A comparison with APACHE III and SAPS II. *Inflammation research : official journal of the European Histamine Research Society [et al]* 2004; **53**(4): 158-63.

49. Yan Y, Hu Y, Wang X, et al. The predictive prognostic values of serum interleukin-2, interleukin-6, interleukin-8, tumor necrosis factor-alpha, and procalcitonin in surgical intensive care unit patients. *Annals of translational medicine* 2021; **9**(1): 56.
